# Shifts in Antarctic Intermediate Water properties coincide with atmospheric CO_2_ rise across the Mid-Brunhes Event

**DOI:** 10.1126/sciadv.ady4567

**Published:** 2026-04-29

**Authors:** Raúl Tapia, Sze Ling Ho, Dirk Nürnberg, A. Nele Meckler, Yoshiyuki Iizuka, Ralf Tiedemann

**Affiliations:** ^1^ Institute of Oceanography, National Taiwan University, Taipei, Taiwan.; ^2^ GEOMAR Helmholtz Center for Ocean Research Kiel, Kiel, Germany.; ^3^ Department of Earth Science, University of Bergen, Bergen, Norway.; ^4^ Bjerknes Centre for Climate Research, Bergen, Norway.; ^5^ Institute of Earth Sciences, Academia Sinica, Taipei, Taiwan.; ^6^ Alfred Wegener Institute Helmholtz Center for Polar and Marine Research, Bremerhaven, Germany.

## Abstract

Antarctic Intermediate Water (AAIW) is key to the global carbon cycle, yet its influence on past atmospheric CO_2_ changes remains unclear. Using multiproxy reconstructions from the data-poor Pacific sector of the Southern Ocean, we examine interglacial AAIW variability in its source region across the Mid-Brunhes Event (MBE), a major CO_2_ transition. While surface temperatures remained stable over 600 thousand years, post-MBE AAIW became warmer and saltier, possibly due to reduced iceberg-derived freshwater input. In contrast, colder, fresher pre-MBE AAIW and enhanced thermal stratification may have promoted greater CO_2_ uptake and storage. The post-MBE declining sequestration capacity of AAIW, coinciding with rising atmospheric CO_2_, suggests intermediate waters played a critical role in modulating CO_2_, challenging the view that changes in bottom-water processes alone controlled this key climatic transition.

## Teaser

Meltwater-driven shifts in Antarctic Intermediate Water weakened ocean carbon storage across the Mid-Brunhes Event.

## INTRODUCTION

The Southern Ocean (SO) is a critical conduit between the deep ocean and the atmosphere in the global carbon cycle (*1*, *2*). In this region, CO_2_-rich deep waters upwell, release CO_2_ to the atmosphere, and subsequently sink to replenish the deep-ocean CO_2_ reservoir. While changes in bottom-water formation and properties have been extensively studied for their role in modulating glacial-interglacial fluctuations in atmospheric CO_2_ concentration (*1*, *2*), Antarctic Intermediate Water (AAIW) has received comparatively little attention. Formed in the SO and ventilating the 500- to 1500-m-depth range globally, AAIW plays a key role in the deep-ocean ventilation (*3*), accounting for 82% of the northward return flow of upwelled SO waters (*4*) as the upper branch of the Antarctic meridional overturning circulation.

AAIW forms in the Polar Frontal Zone, between the Subantarctic Front (SAF) and the Polar Front (PF), where its CO_2_ uptake is primarily governed by surface temperature, salinity, and atmospheric exposure (*2*, *5*, *6*), with additional biological influences (*7*). Two key mechanisms control the temperature and salinity of AAIW: (i) wind-driven Ekman transport (*8*–*10*) and (ii) buoyancy changes driven by surface heating and freshwater inputs as waters flow northward before subducting (*11*). Although their relative influence remains uncertain, stronger winds and greater freshwater input generally produce cooler, fresher AAIW (*4*, *8*, *12*). The latitudinal extent of subducting surface water, characterized by potential density classes between ~27 and 27.3 kg m^−3^ (*13*–*15*), determines the magnitude of air-sea CO_2_ exchange. Once subducted, the CO_2_ content of AAIW is influenced by the vertical thermal gradient with overlying Subantarctic Mode Water (SAMW), which regulates the upper-ocean stratification and carbon retention (*1*, *16*).

Although the South Pacific is the principal source region of AAIW (*11*, *13*), the scarcity of long, continuous sedimentary records has limited reconstructions of its physical properties, past variability, and role in CO_2_ outgassing. To address this knowledge gap, we present a reconstruction of the upper Subantarctic Pacific’s hydrographical properties extending across the Mid-Brunhes Event [MBE; ~424 to 478 thousand years ago (ka)], which marks a shift to systematically higher interglacial CO_2_ levels (~30– to 40–part-per-million increase) (*17*, *18*). This rise reflects a shift in the average state of interglacial climates and is not accompanied by major changes in glacial CO_2_ concentrations, which remained comparatively stable across this transition.

The interglacial shift in atmospheric CO_2_ levels across MBE has been attributed to a fundamental reorganization of the processes regulating CO_2_ emissions and drawdown, including a southward shift of the Southern Westerly Winds (SWW), which enhanced upwelling of CO_2_-rich deep waters during post-MBE interglacials (*19*), and insolation-driven changes in sea ice extent and surface water density, which reduced the formation of Antarctic Bottom Water (AABW) after the MBE (*20*). While these processes are typically discussed in the context of deep-ocean ventilation (*21*–*24*), the formation of both deep and intermediate waters is controlled by high-latitude processes, such as sea-ice formation, buoyancy forcing, and Ekman transport. Despite playing a key role in ocean ventilation, the potential contribution of intermediate water formation to the CO_2_ transition across the MBE remains poorly explored.

**Fig. 1.**
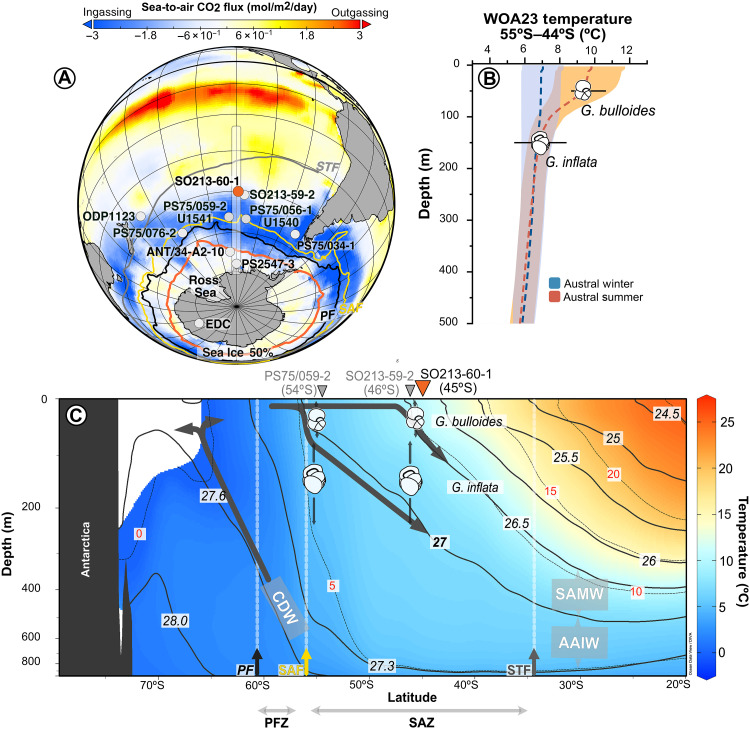
Core location and oceanographic setting. (**A**) Sea-to-air CO_2_ flux based on the SeaFlux product (*87*). Colored lines from north to south depict the location of the modern Subtropical Front (STF; gray), SAF (yellow), the PF (black) (*88*), and the 50% sea ice extent (red line) (*87*), respectively. Core SO213–60–1 (45°S; 119°W) is marked by orange dot; reference sites are in gray: ODP Site 1123 (41°S; 171°W), SO213–59–2 (46°S; 116°W), PS75/059–2 and IODP Site U1541 (54° S; 125° W), PS75/056–1 and IODP Site U1540 (55° S; 114°W), PS75/034–1 (54° S; 80° W), PS75/076–2 (56°S, 156°W), ANT34/A2–10 (67°S; 125°W), PS2547–3 (71°S; 120°W), and the Antarctic ice core EDC (75°S; 123°E). The dashed rectangle outlines the oceanographic profile in (C). (**B**) Vertical distribution and calcification depths of *G. bulloides* and *G. inflata* across 54°S to 44°S inferred from Mg/Ca derived temperatures from cores SO213–60–1, SO213–60–2, SO213–59–1, PS75/056–1, and PS75/059–2, in agreement with plankton net observations at similar latitudes in Atlantic sector of the SO (*68*). Seasonal average temperatures (austral summer and winter) (*71*) between 55° and 44° S are indicated by dashed lines; shading indicates the range between maxima and minimum values. (**C**) South-to-north cross section of temperature in the upper 800 m (color scale and red contour labels on dashed black lines), overlaid with potential density contours (solid black lines) corresponding to SAMW (26.5–27.0 kg m^−3^), AAIW (27.0–27.3 kg m^−3^), and Circumpolar Deep Water (CDW; >27.3 kg m^−3^). Horizontal arrows indicate the latitudinal extent of the Polar Frontal Zone (PFZ; between PF and SAF) and the Subantarctic Zone (SAZ; between SAF and STF). Vertical black arrows indicate calcification depth ranges of the foraminiferal species studied. EDC, EPICA Dome C.

To address this, we reconstruct the temperature and salinity structure of AAIW and SAMW over the past 600 thousand years (kyr) to evaluate how stratification, surface buoyancy, and subduction dynamics evolved across the MBE. This reconstruction is based on foraminiferal Mg/Ca ratios, clumped isotopes (∆_47_), and stable oxygen isotopes (δ^18^O) data from sediment core SO213–60–1 (45°S; 119°W; 3471-m water depth; Fig. 1A). This dataset provides previously unidentified insights into the evolution of AAIW and its potential role in the transition toward higher interglacial atmospheric CO_2_ levels post-MBE.

AAIW, characterized by low salinities [34.2 to 34.5 practical salinity unit (PSU)], temperatures of ~2° to 8°C, and densities of ~27 to 27.3 kg m^−3^ (*13*–*15*), was reconstructed using the geochemical signals from *Globorotalia inflata*, a planktonic foraminifera species that calcifies below the thermocline. In contrast, surface waters influenced by SAMW were tracked using *Globigerina bulloides*. This vertical ecological partitioning in the central South Pacific is supported by core-top and downcore data from 54°S (site PS75/059–2) (*25*) and 46°S (site SO213–59–2) (*26*) (Fig. 1, B and C). Between these latitudes, *G. bulloides* resides in surface waters, while *G. inflata* inhabits AAIW at 54°S and the SAMW-AAIW mixing zone at 46°S, near the isopycnal (27 kg m^−3^; Fig. 1C). Over the past 200 kyr, Mg/Ca-derived sea surface temperatures (SST_Mg/Ca_) at 46°S (~10°C) and 54°S (~9°C) fall within the expected SAMW range (~8° to 15°C) north of the SAF (*13*–*15*). Subthermocline temperatures (SubT_Mg/Ca_) at 46°S (~7°C) and 54°S (~4°C) (*25*, *26*) closely match modern AAIW conditions.

## RESULTS

**Fig. 2.**
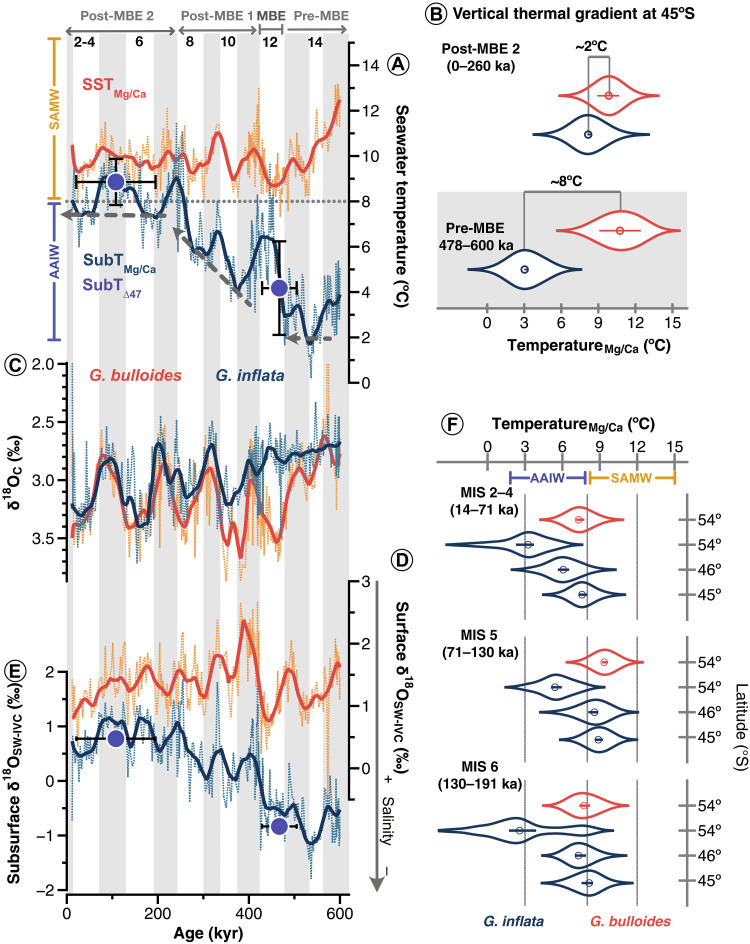
Geochemical data of surface-dwelling *G. bulloides* and subthermocline-dwelling *G. inflata* in the South Pacific. (**A**) Reconstructed surface (SST_Mg/Ca_) and subthermocline temperatures (SubT_Mg/Ca_ and SubT_∆47_). (**B**) Comparison of the vertical thermal gradient (∆*T* = SST_Mg/Ca_ − SubT_Mg/Ca_) at 45°S during post-MBE2 and pre-MBE intervals. (**C**) Oxygen isotopic composition (δ^18^O) of *G. bulloides* and *G. inflata*. (**D**) Reconstructed surface and (**E**) subthermocline relative salinity (δ^18^O_SW-IVC_). SubT_∆47_ and ∆_47_-salinity estimates based on 37 and 34 measurements for post-MBE2 and pre-MBE periods, respectively (see table S1 for details). Filled circles denote mean ∆_47_ estimates; vertical whiskers show the standard error of the mean (2σ), and horizontal whiskers depict the age range of the selected samples (see table S1 for details). (**F**) SST_Mg/Ca_ (red) and SubT_Mg/Ca_ (blue) at 45°S (SO213–60–1), 46°S (SO213–59–2), and 54°S (PS75/059–2) for MIS 2 to 4, MIS 5, and MIS 6, respectively. Note the similarity between SST_Mg/Ca_ at 54°S and SubT_Mg/Ca_ at 45°S to 46°S over time. Violin plots show the distribution of values; lines within violins depict 95% confidence interval, and symbols depict median values. Thick lines in (A) to (D) represent LOESS smoothing curves, fitted with a first-degree polynomial and a 3% smoothing width, achieving a coefficient of determination (*R*^2^) of greater than 0.8.

At 45°S (SO213–60–1), SST_Mg/Ca_ suggests relatively stable surface ocean conditions over the past 500 kyr, with no long-term trend beyond glacial-interglacial oscillations, except for a warmer interval between 500 and 600 ka (Fig. 2A). In contrast, both Mg/Ca and ∆_47_ thermometries reveal a pronounced post-MBE rise in subthermocline temperatures (SubT), with ~5°C of warming across the MBE (Fig. 2A and table S1). The strong agreement between these independent proxies supports the robustness of this temporal shift in temperature (see Materials and Methods). SubT evolution can be divided into three phases: (i) a cold pre-MBE period (~600 to 478 ka), averaging ~4°C; (ii) a transitional post-MBE 1 period (~424 to 260 ka), marked by progressive warming; and (iii) a post-MBE 2 period (~260 to 0 ka), with stabilized SubT averaging ~9°C (Fig. 2A and table S1). The vertical thermal gradient (∆*T*) decreased by ~6°C, from ~8°C in the pre-MBE interval to ~2°C in post-MBE 2 (Fig. 2B).

The δ^18^O values of *G. bulloides* and *G. inflata* range from 2.5 to 3.9 per mil (‰) and 1.5 to 3.7‰, respectively, with the lowest values and smallest glacial-interglacial amplitudes occurring before the MBE (Fig. 2C). Trends in sea surface and subthermocline salinities, inferred from ice volume-corrected seawater δ^18^O (δ^18^O_SW-IVC_; Fig. 2, D and E), show distinct patterns: Surface salinity remains relatively stable, except for a marked increase during Marine Isotope Stage (MIS) 11, while subthermocline salinity exhibited a long-term increase, transitioning from fresher pre-MBE to saltier conditions after the MBE (Fig. 2, D and E).

## DISCUSSION

### *G. inflata* as a recorder of mode water-AAIW in the South Pacific

*G. inflata* is a reliable proxy for AAIW variability in the South Pacific, as it calcifies below the thermocline (Fig. 1, B and C) (*25*, *26*). SubT_Mg/Ca_ recorded by *G. inflata* at 54°S (*25*), 46°S (*26*), and 45°S (this study) are significantly cooler than SST_Mg/Ca_ recorded by *G. bulloides* at the same sites (Welch’s *t* test, *P* < 0.001) (Fig. 2F and table S2), confirming that the species records different water masses (fig. S1). Notably, SST_Mg/Ca_ at 54°S closely matches SubT_Mg/Ca_ at 45°S, as shown by their overlapping distributions and near-identical median values (Welch’s *t* test, *P* > 0.5; table S2).

Downcore records show that AAIW-characteristic temperatures (≤8°C) at 45°S to 46°S only occur when similarly cold temperatures are present in the surface ocean at 54°S (Fig. 2F). This suggests that both reflect the same water mass, AAIW, sampled at different depths and latitudes, consistent with the modern subduction of AAIW density classes (~27 to 27.3 kg m^−3^) near the SAF (Fig. 1, A and C) (*11*, *27*). The persistence of this latitudinal pattern over the past 200 kyr (Fig. 2F and fig. S1), and similar trends in deeper-dweller species (i.e., *Globorotalia truncatulinoides* and *Globorotalia crassaformis*; fig. S2) supports the use of *G. inflata* at SO213–60–1 as a robust recorder of intermediate waters properties. Assuming the same relationship holds true beyond 200 ka, the observed warming and salinization of subthermocline waters across the MBE at 45°S (Fig. 2, A, D, and E) likely reflect a shift toward warmer, saltier AAIW sourced near the SAF.

### Wind-driven Ekman transport and sea ice as drivers of AAIW

**Fig. 3.**
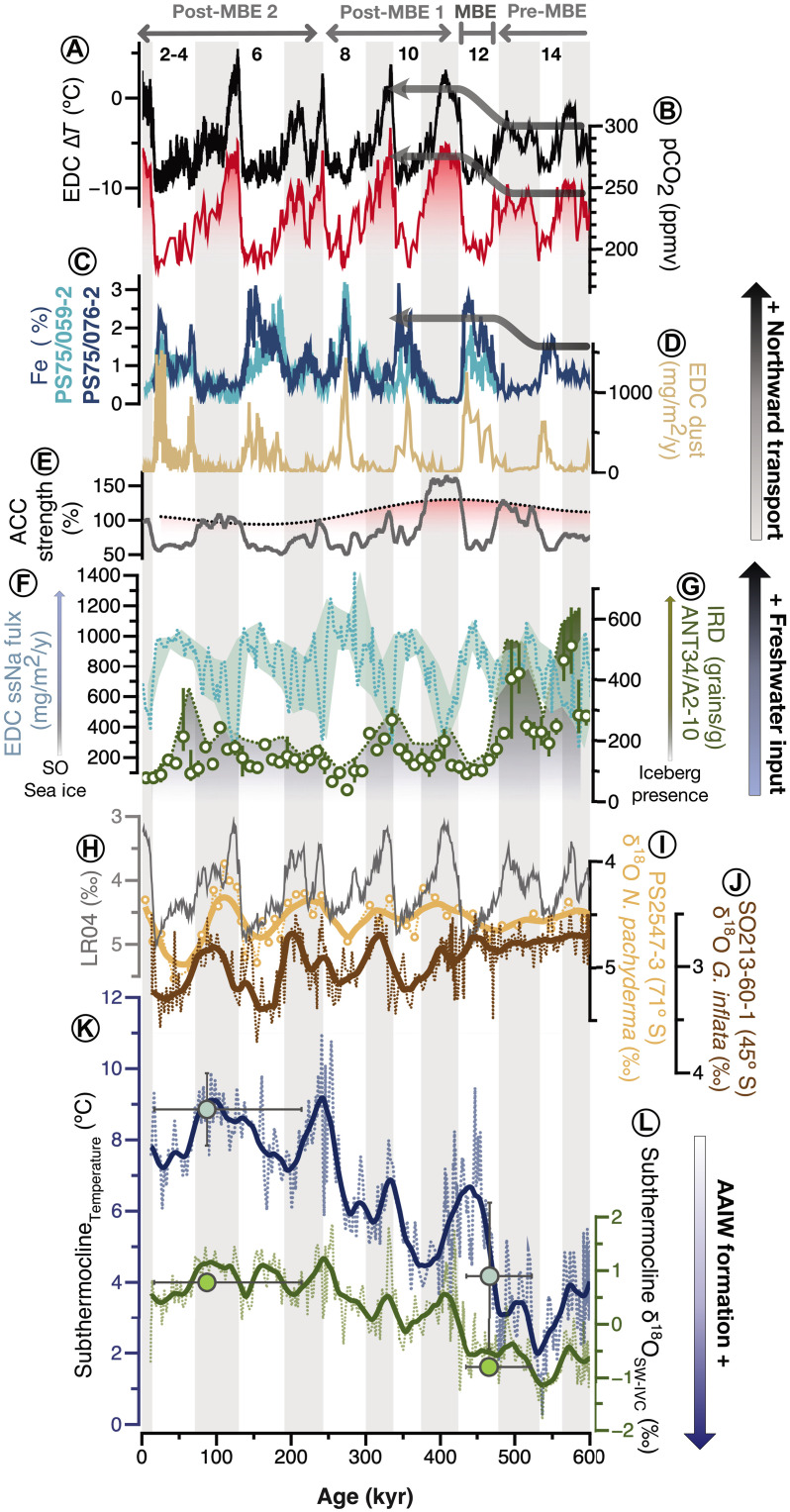
SO dynamics over the last 600 kyr. (**A**) EDC ice core ∆*T* record, reflecting Antarctic air temperature variability (*89*). (**B**) EDC ice core *p*CO_2_ record (*17*). (**C**) Iron flux at core sites PS75/059–2 (54°S) and PS75/076–2 (56°S) (*31*). (**D**) Iron flux from the EDC ice core. (**E**) Average strength of the Antarctic Circumpolar Current (ACC) between 54° and 55°S, relative to the Holocene (*46*). (**F**) Sea-salt sodium (ssNa) flux from EDC ice core, used as a proxy for SO sea ice extent (*36*). (**G**) Iceberg-rafted debris (IRD) concentrations at site ANT34/A2–10 (67°S), indicating episodes of continental ice discharges (*41*). (**H**) LR04 benthic δ^18^O stack (*85*), showing glacial-interglacial variability associated with global ice volume changes. (**I**) δ^18^O record based on *Neogloboquadrina pachyderma* from the Amundsen Sea (PS2547–3) (*47*). (**J**) δ^18^O record based on *G. inflata* at 45°S (this study). Both records [(I) and (J)] show a shift from lower values and reduced amplitude before the MBE to higher values and greater amplitude afterward, suggesting a stronger freshwater component in the δ^18^O signal. (**K**) Subthermocline temperatures (SubT_Mg/Ca_ and SubT_∆47_) and (**L**) subthermocline salinities (δ^18^O_SW-IVC_) at 45°S in the South Pacific, reconstructed from foraminiferal Mg/Ca (lines) and ∆_47_ (symbols). Thick lines in (I) to (L) represent LOESS smoothing curves, fitted with a first-degree polynomial and a 3% smoothing width, achieving a coefficient of determination (*R*^2^) of greater than 0.8. Thick arrows highlight key environmental and climatic transitions, including shifts in freshwater input and AAIW formation. y, year.

Proxy records from the Southeast Pacific (0°S to 54°S) associate colder, fresher, more oxygenated, and vertically expanded intermediate waters with intensified wind-driven Ekman transport during glacial periods driven by stronger SWW (*25*, *26*, *28*–*30*). Notably, beyond the glacial-interglacial contrast, a distinctive post-MBE shift emerges: After the MBE, interglacial atmospheric CO_2_ concentrations and Antarctic temperatures increased (Fig. 3, A and B), and glacial iron fluxes reached higher peak values (Fig. 3, C and D).

Although dust records primarily reflect glacial-interglacial variability and do not resolve wind strength during interglacials, a mean southward migration of the westerlies across the MBE (*19*), indicating broader circulation shifts that could have influenced Ekman transport and intermediate water formation. Under this framework, a post-MBE southward migration and strengthening of the SWW belt (*19*, *31*) would be expected to promote long-term cooling and freshening at 45°S. However, this expectation contrasts with our proxy data, which show instead warmer, saltier subthermocline waters after the MBE and cooler, fresher conditions before it (Fig. 3, C, K, and L). This discrepancy suggests that changes in SWW strength were not the dominant control on AAIW temperature and salinity at this latitude on multiglacial timescales.

Freshwater input plays a critical role in modulating AAIW properties by influencing upper-ocean stratification and water mass transformation (*4*, *12*, *32*, *33*). Freshwater dynamics in the SO are governed by two opposite processes: (i) sea ice formation, which removes freshwater thereby increasing salinity and density; and (ii) melting of sea ice and icebergs, which adds freshwater and lowers surface salinity (*4*, *12*, *32*). Sea ice melt introduces large volumes of freshwater into the Polar Frontal Zone, reducing sea surface salinity and density, and shifting the salinity minimum northward (*33*–*35*). Model simulations confirm this mechanism, showing that freshening, driven by substantial sea ice-derived freshwater input during the Last Glacial Maximum, displaced the AAIW formation farther north (*33*).

Sea-salt sodium (ssNa) fluxes from the Dome C ice core indicate stable interglacial sea ice coverage over the past 800 kyr (Fig. 3F) (*36*). Although local processes such as uplift, transport, and deposition can influence ssNa levels at specific ice core sites, the lack of correlation between the ssNa record and SubT and salinity trends at 45°S (Fig. 3, F, K, and L) suggests that sea ice extent alone did not drive the observed MBE-related changes. Instead, this points to an alternative freshwater source, distinct from sea ice, as the primary driver of subthermocline hydrographic variability at 45°S.

### Calving and ACC as sources of freshwater to AAIW

Icebergs, like sea ice, contribute large volumes of freshwater, accounting for 1300 to 2000 billion tonnes year^−1^ of Antarctica’s freshwater flux (*37*). In the modern South Pacific sector, most icebergs remain south of the PF (~60°S), although some drift beyond the SAF to ~50°S (fig. S3). Thus, increased calving or changes in iceberg melt—driven by iceberg size, air, and water temperatures—could enhance freshwater export into the Polar Frontal Zone (*38*) and influence the precursor waters of AAIW.

**Fig. 4.**
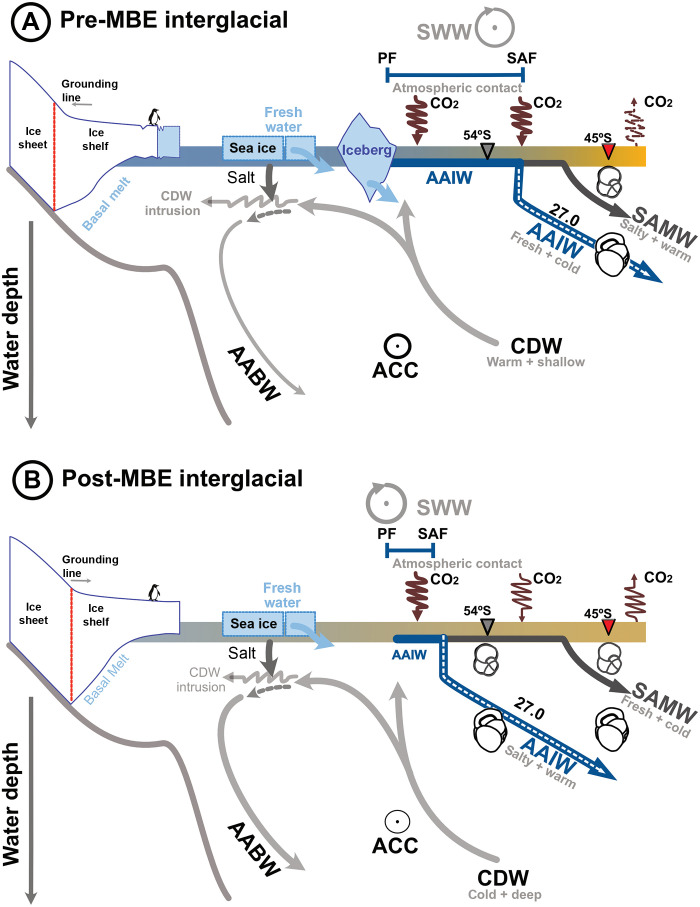
Schematic illustrating the impact of freshwater supply on the AAIW and CO_2_ drawdown across the MBE. (**A**) Interglacial pre-MBE conditions: strong calving by intrusions of warm CDW, upwelled by northward displaced SWW. Enhanced calving promoted ice shelf thinning and iceberg discharge (cf Fig. 3G) increasing freshwater supply (cf Fig. 3I). A stronger ACC (cf Fig. 3E) enhanced the northward transport of fresh water toward AAIW formation zone. These conditions led to the formation of colder and fresher AAIW during the pre-MBE interglacials (cf Fig. 3, K to I), promoting more efficient atmospheric CO_2_ drawdown. In addition, strong stratification increased CO_2_ retention making AAIW a more effective CO_2_ sink. (**B**) Interglacial post-MBE conditions: weaker calving and reduced iceberg discharge, a weakened ACC, led to the formation of a warmer and saltier AAIW with reduced CO_2_ uptake potential. Smaller atmospheric exposure and weaker stratification further contributed to decrease CO_2_ uptake and retention.

In the modern SO, basal melt of Antarctic ice shelves is strongly influenced by incursions of warm Circumpolar Deep Water (CDW) onto the continental shelf (Fig. 4, A and B) (*39*, *40*). These intrusions are facilitated by reduced surface water density, often due to freshwater input from sea ice and iceberg melt, as well as changes in wind patterns that enhance upwelling and CDW access to grounding lines. Once on the shelf, CDW delivers subsurface heat to ice shelf bases, driving melt and promoting iceberg calving (Fig. 4, A and B) (*39*, *40*). At ~45°S, proxy records show colder and fresher subthermocline conditions before the MBE, coinciding with a doubling in median iceberg-rafted debris (IRD) counts at ~67°S during MIS 13 and 15 compared to younger interglacials (core ANT34/A2–10; Fig. 3G) (*41*). This suggests enhanced interglacial iceberg discharge from Antarctica, supplying additional freshwater into the AAIW formation zone, consistent with amplified CDW intrusion and iceberg meltwater release (*42*). Wind-stress shifts may have amplified this chain of processes. Pre-MBE northward-displaced Southern Hemisphere westerlies (*19*) could have boosted interglacial upwelling of warm CDW and, by extension, increased the likelihood of ice-shelf thinning and elevated calving activity (Fig. 4A). After the MBE, a southward migration of the westerlies (Fig. 4B) (*19*) combined with evidence for more stable ice margins and expanded grounding zones (*43*, *44*) likely reduced calving-driven freshwater input.

Freshwater delivery to the AAIW formation zone is also modulated by Antarctic Circumpolar Current (ACC) strength (*38*, *45*), which is driven by wind stress, deep-ocean bathymetry and buoyancy forcing (*11*). As today, the ACC likely played a key role in the past, transporting icebergs from the Ross Sea or farther west (fig. S3) (*38*, *45*). Wang *et al.* (*41*) suggested that the high IRD counts at 67°S before MBE were partly due to a stronger ACC, which advected a large number of icebergs from the Ross Sea. Sortable silt records at ~54°S further support this interpretation, showing that the ACC appears to have been 130 to 150% stronger between MIS 21 and MIS 11 compared to the Holocene (Fig. 3E) (*46*). Beyond eastward transport, the strength of the ACC also influences northward transport through wind-driven Ekman processes and density-driven baroclinic adjustments, which modulate both eddy-induced and residual circulation (*11*, *46*). The combination of high iceberg calving rates supplying freshwater and strong ACC exporting it northward led to cooler, fresher subthermocline conditions at 45°S before the MBE, modifying AAIW properties. A larger pre-MBE freshwater influx is further reflected in the muted δ^18^O variability of planktic foraminifera compared to the global ice volume signals (i.e., core site PS2547–3 versus the global benthic stack; Fig. 3, H and I) (*47*). At 45°S, the δ^18^O record of subthermocline dwelling *G. inflata* also exhibits low-amplitude fluctuations before the MBE (Fig. 3J). By contrast, glacial maxima and interglacial minima are evident in surface dwelling *G. bulloides* δ^18^O at 45°S (Fig. 2C). Persistently low δ^18^O values in *G. inflata* before the MBE are consistent with decreased salinity (Fig. 3L), which might have locally overprinted the global ice volume signal (Fig. 3H). These findings support the interpretation of enhanced freshwater export into the AAIW formation region before the MBE.

In summary, iceberg-derived freshwater appears to have been the main freshwater source influencing AAIW properties at ~45°S before the MBE. This freshwater input was likely shaped by variations in ACC strength and iceberg supply. Together, these processes establish a coherent and physically plausible link between Antarctic cryospheric processes and the formation of intermediate waters in the Subantarctic South Pacific.

### Northward shift in AAIW formation and enhanced solubility-driven CO_2_ sequestration

In addition to modifying the temperature and salinity of the precursor (surface water mass) and AAIW, cryospheric freshwater input influences both CO_2_ solubility and concentration. A surface ocean ~4°C cooler before the MBE would have enhanced CO_2_ solubility by 20 to 26% (fig. S4A) (*48*, *49*), thereby increasing AAIW’s capacity to absorb atmospheric CO_2_. While lower interglacial atmospheric CO_2_ concentrations [~30 parts per million by volume (ppmv)] before the MBE (*48*, *49*) may have limited the CO_2_ diffusion into the ocean, Henry’s law (*50*) suggests that this would reduce uptake by only ~10%. Additionally, lower pre-MBE salinities would have further increased surface ocean CO_2_ solubility, although precise quantification remains challenging (see the Supplementary Materials).

The latitudinal extent of air-sea exchange also affect CO_2_ uptake (*5*). In the modern South Pacific, AAIW absorbs atmospheric CO_2_ over a ~4° latitudinal range between the PF and the SAF before subducting (Fig. 4, A and B). Our records show that SubT_Mg/Ca_ at 45°S over the past 200 kyr is comparable to the SST_Mg/Ca_ at 54°S (Fig. 2F). However, before the MBE, SubT_Mg/Ca_ at 45°S was substantially lower and more similar to SubT_Mg/Ca_ at 54°S (~3°C; Fig. 2, B and F). We attribute these low SubTs at 45°S to a marked shift in AAIW formation. Assuming a constant subduction angle and that the 27 kg m^−3^ isopycnal intersected the calcification depth of *G. inflata* at 45°S (~130 m), the isopycnal outcrop at the air-sea interface would have occurred ~7° in latitude farther north before the MBE (fig. S4B). This northward shift would have expanded the area of AAIW-atmosphere interaction by approximately threefold (Fig. 4A and fig. S4B), enhancing CO_2_ uptake through a more efficient solubility pump (fig. S4A). Additionally, weaker winds blowing between the PF and the SAF before the MBE (*19*) may have limited the supply of CO_2_-rich deep water to the surface via upwelling and instead favoring the upwelling of shallower, relatively CO_2_-depleted waters, further enhancing net CO_2_ uptake (Fig. 4A). As the AAIW typically subducts at the SAF (*11*, *13*), our calculation implies a ~7° northward migration of the SAF before the MBE. This interpretation is consistent with alkenone-based SST (*51*) and diatoms (*52*) records from the southeast and central South Pacific, which indicate a similar northward shift in oceanographic fronts across the MBE.

### Upper-ocean stratification enhanced pre-MBE CO_2_ retention

Before the MBE, lower temperatures and salinities, as well as greater atmospheric contact (Fig. 4A), would increase CO_2_ uptake capacity of the AAIW formed in the South Pacific. The ability of AAIW to store CO_2_ also depends on its retention within the ocean interior during northward transits. This retention is strongly influenced by upper-ocean stratification, a condition where denser water lies beneath lighter waters, forming a stable vertical density gradient that inhibits vertical mixing and limits CO_2_ outgassing by restricting the upward transport of deeper carbon-rich waters. Temperature and salinity exert opposing effects on seawater density: warming decreases density, while increasing salinity increases it. In our study area, stratification is largely governed by thermal effects, which account for more than 90% of the density variability (see the Supplementary Materials). The strong pre-MBE thermal gradient (~8°C), compared to the much weaker post-MBE 2 gradient (~2°C) (Fig. 2B), thus suggests a highly stratified Subantarctic Pacific allowing AAIW to efficiently retain its high CO_2_ load.

Our δ^18^O_SW-IVC_ record indicates that the subsurface cooling was accompanied by freshening (Fig. 2, A, C, and E), potentially affecting surface-subsurface density contrast. However, quantitative salinity estimates based on δ^18^O_SW_ are highly uncertain in the subantarctic Pacific, as existing δ^18^O_SW_-salinity relationships are derived from other basins (*53*). When applied to the observed δ^18^O_SW-IVC_ shift across the MBE, these equations imply a salinity change of 3.5 to 7 PSU, an implausible large shift that would require pre-MBE AAIW source waters to be fresher than modern Antarctic Surface Waters (~33.8 PSU) (table S3; see the Supplementary Materials for details).

Due to these limitations, we used regional T-S profiles from World Ocean Atlas to assess whether subsurface freshening could have offset the thermally driven pre-MBE stratification (Fig. 2B and fig. S5). Even with 0.5 PSU decrease, stratification remained nearly three times higher than post-MBE levels, and reducing it fully would require >1.2 PSU freshening, exceeding modern-day regional salinity gradients and unsupported by proxy and hydrographic data (table S4; see the Supplementary Materials).

Together, these results support the dominant role of temperature in governing upper-ocean density structure in our study area. The stronger pre-MBE thermal gradient (~8°C at 45°S) thus provides a diagnostic indicator of a highly stratified Subantarctic Pacific (Fig. 2B). This steeper vertical gradient likely reflects markedly colder subsurface conditions, coupled with a stronger southward subtropical inflow that elevated SSTs (Fig. 2A) (*54*).

Because subsurface waters at our site are ventilated from the SAF region (Figs. 1C and Fig. 2F), the vertical thermal gradient at our study site likely approximates the latitudinal SST contrast between the SAF and our study site. This meridional SST gradient determines the density contrast between subantarctic and subtropical water masses, which, in turn, modulates the strength of the ACC fronts (*46*). Within this framework, the ~6°C reduction in meridional SST gradient across the MBE implies a substantial weakening of the ACC intensity, consistent with long-term decline of ACC frontal intensity in the South Pacific (*46*). These changes across the MBE may represent a later-stage adjustment within the broader reorganization of SO frontal structure that began with the Polar Frontal Zone migration at ~770 ka during the Mid-Pleistocene Transition (*52*), rather than a discrete, final reorganization event.

Increased stratification before the MBE enabled AAIW to retain its high CO_2_ load as it sank beneath the Pacific Gyre. This effect may have extended beyond mid-latitudes, as high stratification is also suggested for the Eastern Equatorial Pacific before the MBE (*55*), pointing to a basin-scale impact on CO_2_ sequestration. Under these conditions, the combination of cooler temperatures, lower salinities, greater atmospheric exposure, and improved CO_2_ retention likely made AAIW a particularly effective CO_2_ sink during this interval.

### Role of pre-MBE AAIW in interglacial carbon cycle

The inferred post-MBE decline in AAIW-mediated CO_2_ sequestration coincides with the transition to higher interglacials CO_2_ levels (Fig. 3B). Although our reconstruction indicates vigorous AAIW formation during both glacial and interglacial periods before the MBE (Fig. 3, K and L), its glacial enhancement likely had limited impact on atmospheric CO_2_, in contrast to its more pronounced role during interglacials. This asymmetry in CO_2_ response arises from fundamental physical and chemical constraints that differ between these climatic states.

Relative to glacial conditions, interglacial SO stratification was reduced and overturning circulation was more active (*1*, *56*), likely allowing for more efficient ventilation of subsurface waters. These conditions enhanced air-sea CO_2_ exchange and increased the ocean’s capacity to release previously sequestered carbon. In this well-ventilated regime, variations in SO circulation, sea ice extent, and frontal system positions had a direct and amplified impact on atmospheric CO_2_ levels (*20*). As a result, modest changes in ocean ventilation could alter CO_2_ uptake and storage (*20*, *21*, *23*, *24*), making intermediate water a potentially strong key modulator of interglacial carbon variability.

In contrast, glacial periods were characterized by strong stratification and limited deep-ocean ventilation in the SO (*1*). A more efficient biological pump, driven by enhanced nutrient utilization and elevated dust-borne iron (*7*, *57*), pushed the ocean toward its maximum carbon storage capacity (*56*). In this state, the potential for further atmospheric CO_2_ drawdown was restricted, even in the presence of intensified AAIW formation. Thus, the post-MBE decline in intermediate-water formation likely played a more prominent role in modulating interglacial, rather than glacial, CO_2_ levels.

### AAIW’s role in carbon storage may be greater than previously thought

One leading hypothesis proposes that increased AABW formation contributed to the lower atmospheric CO_2_ levels before the MBE (*20*). However, more recent model-based studies show that changes in abyssal water formation alone were insufficient to account for the full CO_2_ increase across the MBE (*21*, *23*, *24*), implying that additional mechanisms must have been involved. Our findings suggest that enhanced intermediate-depth carbon storage may represent one such mechanism.

Our reconstruction indicates that, before the MBE, AAIW was colder and fresher due to enhanced upstream formation process and ventilation at the SAF. The enhanced AAIW formation during the pre-MBE period was likely a result of more ice-berg-related freshwater inputs, as evidenced by elevated IRD counts in the polar zone. Notably, high freshwater input can inhibit the formation of AABW (*40*, *58*, *59*), and proxy evidence from the Antarctic sector supports a weak AABW formation during the pre-MBE period (*52*, *60*). These findings illustrate that the two limbs of the SO overturning circulation, the intermediate and abyssal branches, can respond in opposite ways to the same forcing.

Together, our results imply that intermediate waters such as AAIW may have played a more substantial, and previously underrecognized, role in modulating ocean-atmosphere CO_2_ exchange across the MBE transition, complementing rather than contradicting the established importance of AABW dynamics.

## MATERIALS AND METHODS

### Sediment cores and sample preparation

The 673-cm-long sediment core SO213–60–1 (44°57.83′S; 119°33.07′W; 3471-m water depth) was retrieved from the eastern flank of the East Pacific Rise during leg 2 of the SOPATRA cruise using a gravity corer (Fig. 1A) (*61*). The core was sampled at 2-cm intervals, freeze-dried, weighed, washed through a 63-μm sieve, and dried at 50°C. Samples were dry sieved and separated into multiple size fractions. Benthic foraminifera were picked from the 315- to 400-μm fraction, while planktic foraminifera were picked from the 315- to 355-μm fraction.

The top 0 to 1 cm of multicore SO213–60–2 (26 cm long), collected at the same location, underwent the same processing. Foraminiferal material from this core-top was used for Mg/Ca calibration validation and radiocarbon dating. Radiocarbon dating, previously reported by Molina-Kescher *et al.* (*62*), was performed on 1.2 mg of mixed planktonic foraminiferal material (KIA45890) at the Leibniz-Laboratory for Radiometric Dating and Stable Isotope Research, Christian-Albrechts University of Kiel (Germany), using accelerator mass spectrometry (AMS). The radiocarbon age was converted to calendar age using the software CALIB 7, applying a ∆*R* correction of 560 years.

### Foraminifera selection for geochemical analyses

To establish the stratigraphy of core SO213–60–1, we measured stable oxygen isotopes (δ^18^O) on three to eight specimens of benthic foraminifera *Uvigerina peregrina* per sample (311 samples). For δ^18^O and trace metal (Mg/Ca) analyses as proxies for upper ocean conditions, we hand-picked ~30 specimens per sample of the planktic species *G. bulloides* (336 samples) and *G. inflata* (299 samples) from the entire gravity core SO213–60–1 and the top 1 cm of multicore SO213–60–2. For *G. inflata*, heavily encrusted specimens (with smooth surface and shiny appearance) were excluded as their Mg/Ca ratios may be biased (*63*). We also strived to pick only specimens with similar encrustation levels to minimize any systematic shifts in the *G. inflata* proxy records. Additionally, ~35 specimens of *G. inflata* were picked from selected depths (see table S1 and the “Clumped isotope thermometry” section) of core SO213–60–1 for clumped isotope (Δ_47_) analyses.

### SEM of foraminifera

To assess the encrustation and the overall preservation state of foraminiferal tests, we examined scanning electron microscopy (SEM) images of *G. inflata* from selected depths across the core. Tests were placed in the SEM vacuum chamber without coating. Micrographs were obtained using JEOL FE-SEM JSM-7100F (Academia Sinica, Taiwan), operated at 10 kV and 100 pA under low-vacuum condition (50 Pa). SEM images show consistent encrustation levels throughout the core (fig. S6), without any apparent shift across the MBE. This suggests that the substantial shifts in Mg/Ca and δ^18^O are not due to microstructural changes in the tests of *G. inflata*. Additionally, foraminiferal tests exhibit well-preserved wall structures with no evidence of significant dissolution or diagenetic alteration, further supporting the reliability of Mg/Ca reconstructions.

### Clumped isotope thermometry

Sample preparation and cleaning for clumped isotope (∆_47_) analysis was performed according to the protocol of Meinicke *et al.* (*64*). Briefly, the samples were crushed and then sonicated in water and methanol repeatedly, dried, and separated in aliquots of 86 to 105 μg (9 to 14 aliquots per depth; table S1). The analysis was performed using a Thermo Scientific MAT 253 Plus mass spectrometer coupled to a Kiel IV carbonate preparation device (Thermo Fisher Scientific, Bremen, Germany) at the University of Bergen (Norway). Organic contaminants were removed using a Porapak trap held at 50°C. Δ_47_ data were converted to the InterCarb Carbon Dioxide Equilibrium Scale (I-CDES) scale (*65*) using community standards ETH 1–3 in a moving average window approach. Average ∆_47_ data were converted into temperature estimates using the planktic foraminifera-based calibration proposed by Meinicke *et al.* (*66*), updated to the I-CDES scale by Meinicke *et al.* (*64*)

$${∆}_{47}=\left(0.0397\pm 0.0011\right)\times 106/T^{2}+\left(0.1518\pm 0.0128\right)$$ (1)

The Δ_47_ data shown in Fig. 2A are median values of >30 replicates per time slice, with the standard error of the mean (2σ) used as the error estimate (table S1).

### Mg/Ca measurements

Planktic foraminifera shells were gently crushed between two methanol-cleaned microscope glass plates, homogenized, and split into subsamples for Mg/Ca and δ^18^O analyses. This approach allows us to minimize artifacts arising from age-depth uncertainties when combining Mg/Ca and δ^18^O data for seawater δ^18^O (δ^18^O_SW_) reconstructions. Mg/Ca subsamples were cleaned following the cleaning protocol of Barker (*67*), with an added reductive cleaning step using hydrazine. Analyses were conducted using an ICP-OES (VARIAN 720-ES) at GEOMAR-Kiel (Germany) (*68*). Instrument performance was monitored using the ECRM752-1 standard (*69*), and long-term analytical precision is ±0.1 mmol mol^−1^ for Mg/Ca ratios. Fe/Ca, Al/Ca, and Mn/Ca ratios were measured alongside Mg/Ca to check for clay contamination and post-depositional Mn-rich carbonate coatings, which can bias Mg/Ca ratios. The Fe/Ca, Mn/Ca, and Al/Ca values were low (≤0.1 mmol mol^−1^), and no strong correlation was observed between these elemental ratios and Mg/Ca, suggesting negligible contamination (fig. S7) (*67*, *70*).

### Calibration selection

Various equations are available in the literature to convert Mg/Ca data into temperature estimates. While the choice of calibration affects absolute temperature values, relative changes remain fairly consistent, as most equations assume an exponential dependence with an approximate Mg incorporation rate of ~9% per degree Celsius. Recently, multivariate calibrations accounting for pH and salinity effects have been developed, such as those from Gray and Evans (*71*). These calibrations show lower thermal sensitivity (~6%/°C). Unfortunately, species-specific multivariate equations are available for only a handful of surface-dwelling foraminifera species and not for *G. inflata* analyzed in this study. Although a generic multispecies calibration is available, it is not designed to provide absolute temperatures (*71*), making direct comparison with climatological data or multiproxy data challenging.

We evaluated thirteen calibrations (seven for *G. bulloides* and six for *G. inflata*; see tables S5 and S6 and figs. S8 and S9), including both univariate and multivariate approaches. Selection criteria included minimizing the difference between reconstructed core-top (SO213–60–2) temperatures and climatological data and ensuring ecologically consistent observed vertical distribution and calcification depth in the SO (*72*); i.e., mean temperatures for the deep-dwelling *G. inflata* should be cooler than those for the surface-dwelling *G. bulloides*.

Among the univariate calibrations, Mashiotta *et al.* (*73*) for *G. bulloides* and Groeneveld and Chiessi (*74*) for *G. inflata* best matched core-top temperatures with climatological values at depths of 0 to 70 m and 70 to 200 m, respectively, during the austral summer (*75*). This calibration pair also yielded lower *G. inflata* temperatures relative to *G. bulloides* (fig. S8) and showed close agreement with ∆_47_-thermometry (fig. S9). Applying the species-specific multivariate calibration to *G. bulloides* resulted in core-top temperatures of ~3°C lower than those from the Mashiotta calibration and ~5°C below climatological surface (0 to 70 m) temperatures, closer to subthermocline values (fig. S8).

Lacking a species-specific multivariate calibration for *G. inflata*, we applied the multispecies approach (table S5). This yielded the same trend, from cold pre-MBE to warm post-MBE 2 period, as ∆_47_-thermometry and univariate Mg/C reconstructions (fig. S9). Despite limitations in absolute temperature estimation, this calibration produced a consistent thermal gradient (~5°C), aligning with that obtained from the univariate calibration Mg/Ca (~4°C) and Δ_47_ (~5°C). This agreement indicates minimal nonthermal influence on *G. inflata* Mg/Ca-derived temperatures (fig. S9).

Due to the unrealistically low *G. bulloides* temperatures obtained from the multivariate calibration approach, even lower than *G. inflata* temperatures, and the lack of absolute calibration for *G. inflata*, we selected the univariate calibrations of Mashiotta *et al.* (*73*) for *G. bulloides* and Groeneveld and Chiessi (*74*) for *G. inflata* as they better reflect vertical ecological structure and modern climatology of the study area.

### Potential effects of salinity and dissolution on Mg/Ca-derived temperatures

In addition to potential biases from clay contamination and Mn-rich carbonate coatings, the Mg/Ca signal in foraminifera can be affected by calcite dissolution, which preferentially removes Mg-rich calcite and lowers the original Mg/Ca ratio (*76*). Although core SO213–60–1 was retrieved from a depth of 3471 m (∆[CO_3_^2−^] ≈ −4 μmol kg^−1^), near the modern calcite saturation horizon (fig. S10), several observation suggest that dissolution effects are minimal: (i) The Mg/Ca ratios of *G. bulloides* and *G. inflata* are generally considered dissolution resistant and reliable for paleotemperature reconstructions (*25*, *26*, *77*). Applying dissolution corrections of 0.1° to 0.2°C per μmol kg^−1^ for *G. bulloides* and 0.4° to 0.6°C per μmol kg^−1^for *G. inflata* (*72*) yielded temperature estimates higher than modern climatology (fig. S10). (ii) The Mg/Ca-derived temperature records of *G. bulloides* and *G. inflata* show distinct temporal trends (Fig. 2A), suggesting that they were not shaped by a common factor, such as dissolution. (iii) Mg/Ca ratios are more sensitive to dissolution than ∆_47_ thermometry (*78*, *79*). The strong agreement between *G. inflata* Mg/Ca and ∆_47_ temperatures across the core implies limited dissolution, particularly for the low pre-MBE Mg/Ca temperatures (Fig. 2A and table S1). (iv) Dissolution would typically produce heavier δ^18^O values due to the preferential preservation of dissolution-resistant calcite, which has higher δ^18^O values (*63*). Therefore, the co-occurrence of low Mg/Ca temperatures and low δ^18^O values in *G. inflata* before MBE, comparable to peak interglacial values, argues against dissolution (Fig. 2, A and B). These findings collectively support a minimal dissolution-induced bias in our Mg/Ca-derived temperatures.

Salinity influences Mg incorporation in foraminiferal shells; therefore, it may be a potential source of bias in the Mg/Ca thermometry (*80*). Studies on this effect, primarily limited to surface-dwelling symbiont-bearing species, suggest that Mg incorporation varies from 3 to 5% per salinity unit depending the species (*71*, *80*). Whether salinity affects subsurface-dwelling, nonsymbiont bearing *G. inflata* is unclear. However, the similarity between uncorrected Mg/Ca temperatures and those corrected for salinity and their agreement with Δ_47_ temperatures (fig. S9) indicates that modern salinity has limited impact on post-MBE 2 Mg/Ca temperatures.

However, our findings suggest that the subthermocline salinity at our study sites was lower before the MBE. To assess the potential effect of this salinity change on our *G. inflata* Mg/Ca-temperature record across the MBE, we performed a sensitivity test by assuming that *G. inflata* has the same Mg/Ca-salinity sensitivity as symbiont-bearing surface dwellers (fig. S11) using the MgCaRB online tool (*71*). We input the mean Mg/Ca value (0.92 mmol mol^−1^) for the pre-MBE time window assuming an age of 471 kyr and varied the salinity parameter in the multiparametric exponential multispecies calibration from +1 to −9 units while keeping the other parameters constant (i.e., ρCO_2_ disequilibrium and alkalinity; table S6). This analysis shows that a salinity decrease of 1 to 3 salinity units, typical for glacial-interglacial timescales, has no substantial impact on our Mg/Ca-based temperature estimates as they agree within error with ∆_47_ temperatures. This remains true for +1 to −7 units of salinity change. In other words, unrealistically large freshening of >7 salinity units would be required to push Mg/Ca-derived temperatures beyond pre-MBE ∆_47_ constraints (fig. S11). Therefore, we conclude that our findings regarding the observed Mg/Ca-based temperature shift across the MBE would still hold true for a freshening of <7 units, assuming that the salinity sensitivity of *G. inflata* Mg/Ca was similar to that of surface-dwelling species.

### Stable oxygen isotope measurements

Benthic foraminifera samples and the planktic foraminifera subsamples obtained from the same initial sample that was divided for both Mg/Ca and isotope analyses (see the “Mg/Ca measurements” section) were ultrasonically cleaned with deionized water and methanol. The isotopic measurements were performed using a MAT 253 mass spectrometer coupled with a Kiel IV Carbonate device (Thermo Fisher Scientific, Germany) at GEOMAR-Kiel (Germany). Isotope values were referenced to the NBS19 standard and reported on the Vienna Pee Dee Belemnite scale. Analytical SEs were ± 0.05‰ for δ^18^O and ± 0.04‰ for δ^13^C. Samples used for ∆_47_ measurements also yielded δ^18^O values (see the “Clumped isotope thermometry” section). These were calibrated to the VPDB scale using the ETH 1–3 carbonate standards, which were also used for ∆_47_ corrections (*81*). δ^18^O values were additionally referenced to NBS18, NBS19, and LSVEC (*64*). δ^18^O results from GEOMAR-Kiel and the University of Bergen agree within analytical uncertainty (fig. S12). Therefore, both sets of δ^18^O data were included in data visualization and calculations.

### Seawater oxygen isotopic composition, δ^18^O_SW_

To assess past seawater salinity, we used paired δ^18^O-Mg/Ca temperatures data from *G. bulloides* and both δ^18^O-Mg/Ca temperatures and δ^18^O-∆_47_ temperature data from *G. inflata*. Seawater δ^18^O (δ^18^O_SW_) was derived from measured planktonic foraminiferal δ^18^O (δ^18^O_C_) and temperatures estimated (both Mg/Ca and ∆_47_) using the equation of Shackleton *et al.* (*82*)

$$\delta^{18}O_{\text{SW}}\;\left(\text{SMOW}\right)=\delta^{18}O_{C}+0.27-\frac{4.38-\sqrt{4.382-4\times 0.1\times \left(16.9-T\right)}}{0.1\times 2}$$ (2)

Because δ^18^O_SW_ reflects both global ice volume and local hydrological changes, we isolated the local hydrological signal by subtracting the ice volume component (IVC), as published by Elderfield *et al.* (*83*), but interpolated to the temporal resolution of core SO213–60–1. This yielded the ice volume-corrected δ^18^O_SW_ (δ^18^O_SW-IVC_), which serves as a qualitative proxy for local salinity changes (Fig. 2, D and E). We refrained from converting δ^18^O_SW-IVC_ to salinity units due to the large uncertainties related to the salinity-δ^18^O_SW_ relationship in the study area and its temporal validity.

### Age control and chronology

The age model of gravity core SO213–60–1 is based on the visual alignment of its *U. peregrina* δ^18^O and the *U. peregrina* δ^18^O record from the core ODP1123 (41°S) (*83*), using AnalySeries 2.0.4 software (fig. S13) (*84*). Although the global δ^18^O stack (LR04) (*85*) stack is more commonly used for age model establishment, we consider that, at our site, the direct comparison with the site ODP1123 is more appropriate. This is due to apparent differences in the temporal patterns between the two records, likely due to a strong regional signal in the ODP1123 record compared to the globally averaged LR04 stack signal. Furthermore, the ODP1123 core provides a mean seawater δ^18^O (δ^18^O_SW_) record based on bottom water δ^18^O-Mg/Ca estimates derived from *U. peregrina* (*83*), which is essential for deriving the δ^18^O_SW-IVC_ (see the “Seawater oxygen isotopic composition, δ^18^O_SW”_ section). Using a single regional reference record helps reduce chronological uncertainties associated with combining multiple records to obtain the δ^18^O_SW_ component. For these reasons and given that both sediment records are from the same basin, we favor ODP1123 over the LR04 as a reference to build our age model. The robustness of our age model is further supported by biostratigraphic markers (*61*), the similar evolution of the *U. peregrina* δ^13^C curves in both cores (SO213–60–1 and ODP1123), and consistency with well*-*dated δ^13^C records from cores PS75/059–2 and PS75/056–1 (fig. S13) (*86*).

## Supplementary Materials

### The PDF file includes:

Supplementary Text S1 and S2 Figs. S1 to S14 Tables S1 to S6 Legend for data S1 References
