# Supplementary material for: Shifts in Antarctic Intermediate Water properties coincide with atmospheric CO2 rise across the Mid-Brunhes Event
Source: Sci Adv. 2026 Apr 29;12(18):eady4567. doi: 10.1126/sciadv.ady4567 (PMC13127585; doi:10.1126/sciadv.ady4567)
Supplement: Supplementary file 1 — Supplementary Text S1 and S2 Figs. S1 to S14 Tables S1 to S6 Legend for data S1 References [file sciadv.ady4567_sm.pdf]

Science Advances
AAAS

Supplementary Materials for

Shifts in Antarctic Intermediate Water properties coincide with atmospheric CO_2 rise across the Mid-Brunhes Event

Raúl Tapia *et al.*

Corresponding author: Raúl Tapia, raultapia@ntu.edu.tw

Sci. Adv. **12**, eady4567 (2026)
DOI: 10.1126/sciadv.ady4567

The PDF file includes:

Supplementary Text S1 and S2
Figs. S1 to S14
Tables S1 to S6
Legend for data S1
References

Other Supplementary Material for this manuscript includes the following:

Data S1

Supplementary Text

1. Thermal and haline contributions to seawater density

   The relationship between temperature, salinity, and potential density can be approximated using the linearized form of the equation of state for seawater (*90*):

$$\Delta\rho = \rho_0(-\alpha\Delta T + \beta\Delta S)$$

   Here ρ_0 is a reference density, α is the thermal expansion coefficient (~2×10^{-4} ºC^{-1}) indicating how density changes with temperature; and β is the haline contraction coefficient (~7.6×10^{-4} PSU^{-1}), indicating how density changes with salinity. ΔT and ΔS are changes in temperature and salinity, respectively. This approximation is appropriate because the temperature and salinity variations in our study area are relatively small, falling within the range where the linear formulation provides reliable first-order estimates.

   Present-day sea surface conditions between 50º and 42ºS along 119º–120ºW span the frontal zone near the modern Subantarctic Front (SAF), a key region for Antarctic Intermediate Water (AAIW) formation (*75*, *91*). This latitudinal band captures the temperature and salinity gradients relevant to our study site in the central South Pacific and offers a spatially constrained view of regional hydrographic conditions. Within this latitudinal range, surface waters (0–100 m) show a southward temperature decrease of 6 ºC and a salinity decrease of 0.13 PSU, resulting in a density increase of approximately 8.91×10^{-4} kg m^{-3} (fig. S14). At depth (100–300 m), smaller variations—a temperature decrease of 5.5 ºC and a salinity decrease of 0.13 PSU—produce a density increase of about 2.15×10^{-4} kg m^{-3} (fig. S14).

   The thermal and haline contributions to the total density change can be estimated as:

$$\Delta\rho = |\alpha\Delta T| + \beta\Delta S = |2 \text{ x } 10^{-4} \, x \, (-6)\,| + (7.6 \text{ x } 10^{-4} \, x \, 0.13)$$
$$= 1.2 \, x \, 10^{-3} + 9.88 \, x \, 10^{-5} = 1.3 \, x \, 10^{-3}$$

   From this, percentage contributions are:

   Temperature = $1.2 \, x \, 10^{-3}/1.3 \, x \, 10^{-3} \approx 92\%$

   Salinity = $9.88 \, x \, 10^{-5}/1.3 \, x \, 10^{-3} \approx 8\%$

   Consequently, temperature accounts for ~92% of the observed density variability in the surface ocean and subsurface ocean, with salinity contributing ~8%. These results confirm that, at these latitudes, upper-ocean stratification is primarily thermally controlled.

2. Estimating the salinity anomalies needed to offset temperature-induced stratification across the MBE

   To assess how much salinity changes would be needed to overcome the ocean stratification caused by temperature across the MBE, we used climatological data from the World Ocean Atlas 2023 (WOA23) (*75*, *91*). Specifically, we examined modern regional T-S profiles from 69.5° S to 30.5° S along 119–120°W (fig. S5), to calculate potential

density. This latitudinal range includes key zones of water mass transformation in the South Pacific, particularly the Polar Frontal Zone (PFZ) and the Subantarctic Front (SAF), where Antarctic Intermediate Water (AAIW) is formed. It captures the vertical and horizontal variability in temperature and salinity relevant to AAIW source regions and their downstream evolution.

Our goal is not to reconstruct detailed temporal salinity changes, but to estimate how much freshening would be needed to weaken vertical stratification. For this, we use present-day spatial variations in temperature and salinity as a guide for past conditions. This is a common and physically grounded approach in oceanography and paleoclimate research, based on the assumption that the key processes shaping salinity patterns today—such as freshwater input, wind-driven surface transport (Ekman transport), and mixing between water masses—also operated in the past. In regions where direct paleosalinity data are scarce or absent, modern spatial patterns provide a valuable way to estimate past hydrographic variability.

To quantify the necessary salinity changes, we applied reconstructed post-MBE temperatures to the T-S profile at 45.5°S to extract the corresponding salinities at two depth intervals: 0–100 m (surface) and 100–200 m (subsurface) (fig. S5a). The estimated salinity ranges at the surface (34.216 ± 0.036 PSU) and subsurface (34.261 ± 0.051 PSU) were then used to calculate potential densities using the Sea Water Equation of State Calculator (TEOS-10 and IES-80) (*92*). These results suggest a reference post-MBE density contrast ($\Delta\rho = \rho_{surface} - \rho_{subsurface}$) of −0.29 ±0.02 kg m^{-3} confirming that the subsurface remains denser than the surface.

To estimate surface salinity during the warmer and saltier pre-MBE period, we overlaid reconstructed pre-MBE upper ocean temperatures onto climatological T-S profiles between 45.5° and 30.5°S (fig. S5b). This comparison suggests that a +1°C warmer pre-MBE surface ocean would be associated with only a minor salinity change from +0.01 to +0.05 PSU (fig S5b and table S4).

A similar approach was applied to estimate subsurface salinity under the cooler, fresher pre-MBE conditions. By applying pre-MBE temperatures to the T-S profiles from 60.5º to 45.5º S (fig. S5c), we estimate a ~5ºC cooling could be associated with a freshening between –0.3 and –0.26 PSU (fig. S5c).

Even under this high salinity contrast scenario of a +1ºC warming and maximum surface salinity (34.25+0.05 PSU; maximum density) and a –5ºC cooling with minimum salinity (34.21–0.3 PSU; minimum density), the resulting pre-MBE density gradient would reach up to –0.73 kg m^{-3}. This value is nearly three times greater than the post-MBE reference gradient, attesting to enhanced pre-MBE stratification under plausible hydrographic extremes. To reduce the elevated pre-MBE stratification to post-MBE levels, a subsurface freshening exceeding 1.2 PSU (table S3) —roughly twice the magnitude of modern-day regional salinity variability—would have been required.

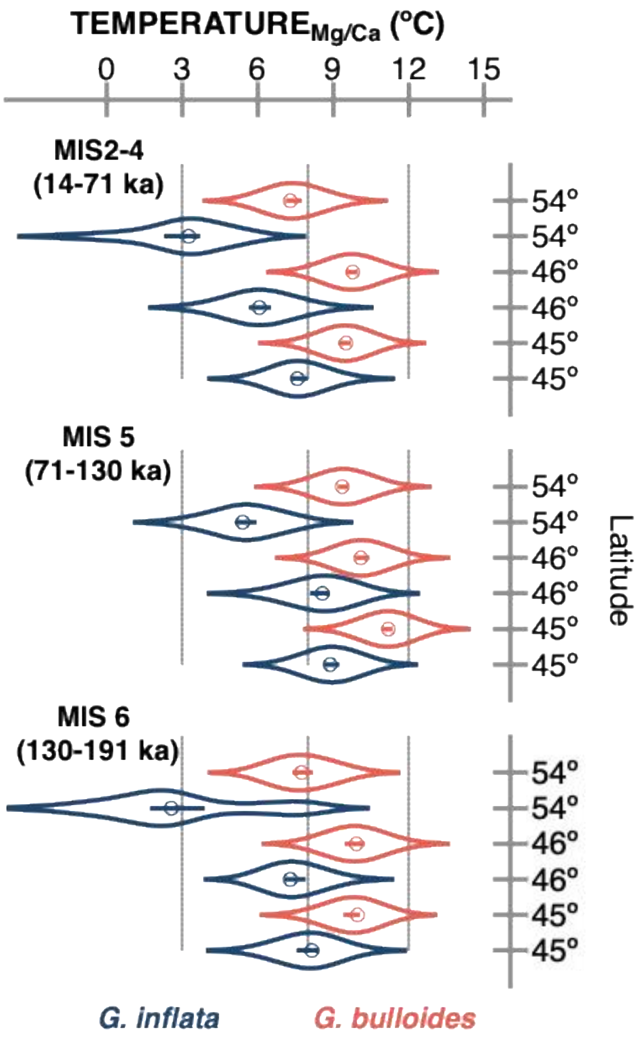

Fig. S1.

Mg/Ca-derived temperatures from *G. bulloides* ($SST_{Mg/Ca}$; red) and *G. inflata* ($SubT_{Mg/Ca}$; blue) at 45ºS (SO213–60–1; this study), 46ºS (SO213–59–2) (*26*) and 54ºS (PS75/059–2) (*25*) for MIS 2–4, MIS 5 and MIS 6 showing consistently higher $SST_{Mg/Ca}$ than $SubT_{Mg/Ca}$ at all sites. Violin plots show the distribution of values; lines within violins depict 95% confidence interval, and symbols depict median values.

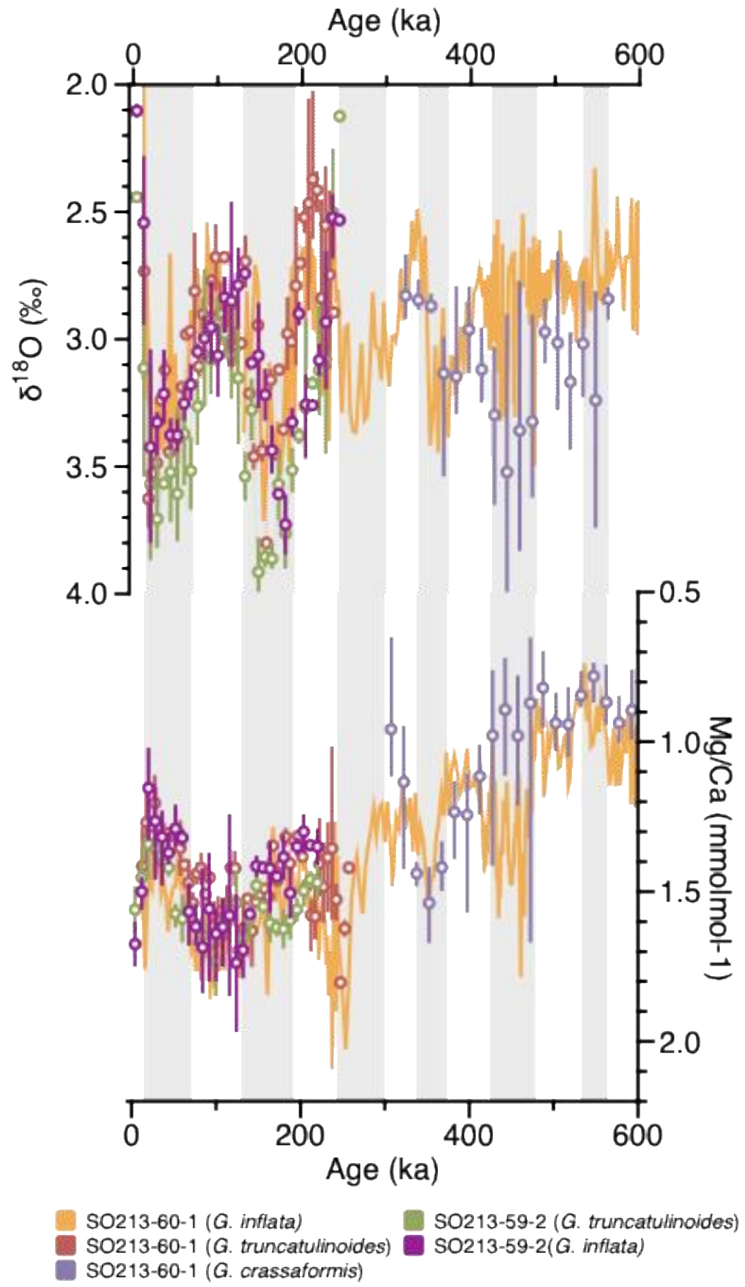

Fig. S2.

Comparison of planktic foraminiferal $\delta^{18}O$ (left axis) and Mg/Ca values (right axis) records from cores SO213-59-2 (46ºS) (*26*) and SO213-60-1 (this study). Colored symbols denote different deep-dwelling species: *G. inflata* (orange, purple), *G. truncatulinoides* (red, green), *G. crassaformis* (light purple). The consistent direction of change across species, along with similar absolute values, supports the interpretation that *G. inflata* reliably captures the intermediate water signal. Gray vertical bars mark interglacial periods. Data points show mean values binned at 10 kyr; whiskers denote the maximum and minimum values within each bin.

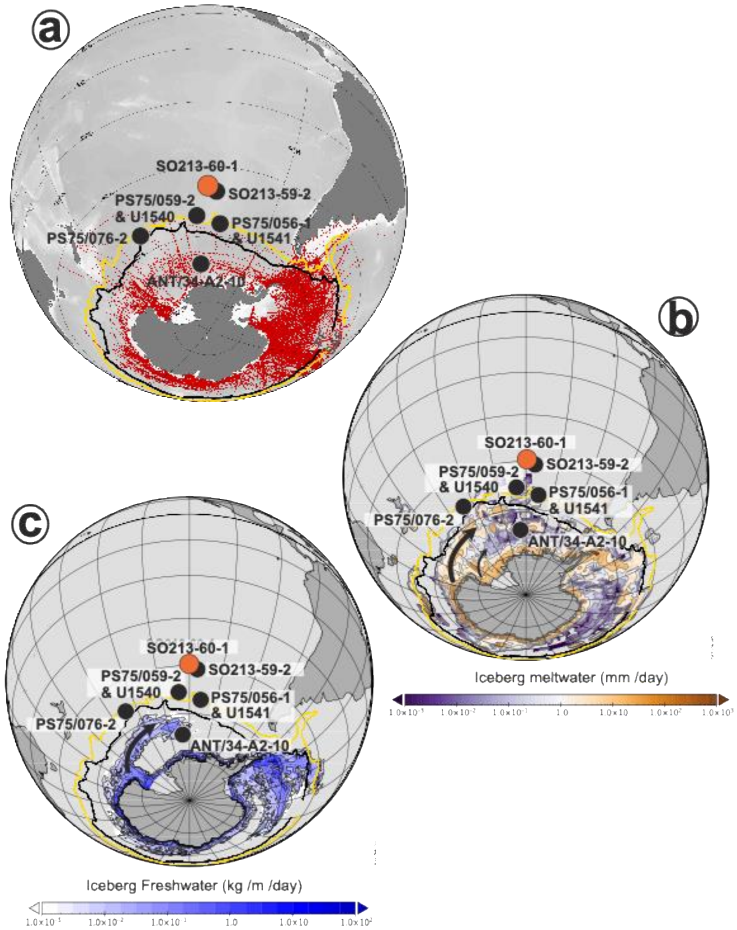

Fig. S3.

a) Iceberg distribution in the Southern Ocean 1977 – 2010 (SCAR database) (*93*). Iceberg meltwater climatology for the late Austral summer (mean values for January and February) based on b) Rackow et al. (*38*) and c) Merino et al. (*45*), showing downstream advection transport of the freshwater by the ACC (black arrows) toward the central South Pacific and north of the Subantarctic Front (SAF). Symbols indicate core site locations, colored lines mark the location of the Polar front (black) and SAF (yellow) (*88*).

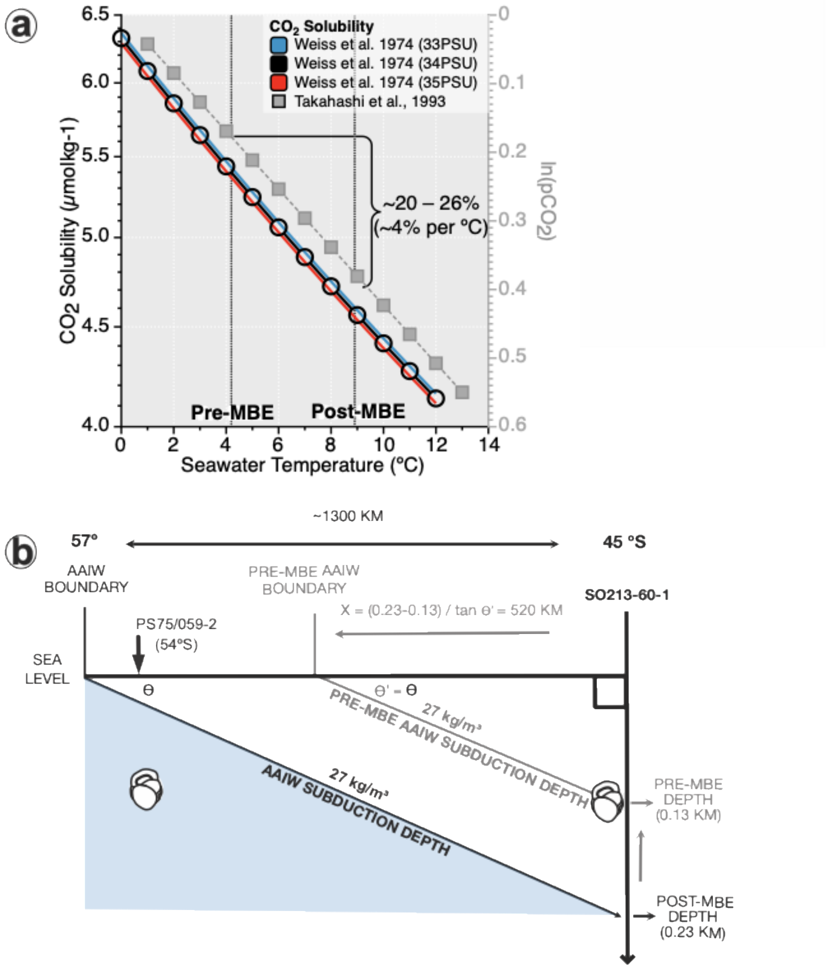

Fig. S4.

(a) Estimated 20–26% decrease in CO_2 solubility after the Mid-Brunhes Event (MBE), attributed to warmer seawater temperatures-Solubility was calculated using the equations of Weiss et al. (*48*) and Takahashi et al. (*49*). While increased salinity after the MBE would further reduce CO_2 solubility, this effect is not included due to the lack of quantitative salinity estimates (see Seawater oxygen Isotopes $\delta^{18}O_{SW}$ section). (b) Sketch of the inferred latitudinal shift in the atmospheric interaction zone of Antarctic Intermediate Water (AAIW) in the South Pacific across the MBE. Today, the AAIW isopycnal reaches ~0.23 km below the surface at core site SO213–60–1 (~45ºS) and intersect the surface near the Subantarctic Front (SAF) at ~57ºS–defining the modern northern limit of AAIW formation, ~1300 km south of core. Pre-MBE, temperature and salinity suggest that AAIW extended to ~130 m, intersecting the calcification depth of *Globorotalia inflata*. Assuming a constant subduction angle (ϴ ≈ ϴ') and that *G. inflata* maintained a similar calcification depth over time, the pre-MBE northern boundary of AAIW is estimated to be ~520 km south of 45ºS (X = 0.1 / tan ϴ' ≈ 520 km ≈ 5º in latitude). This implies a ~780 km (~7º latitude) northward shift in the AAIW outcrop relative to modern conditions, suggesting a threefold increase in atmospheric exposure.

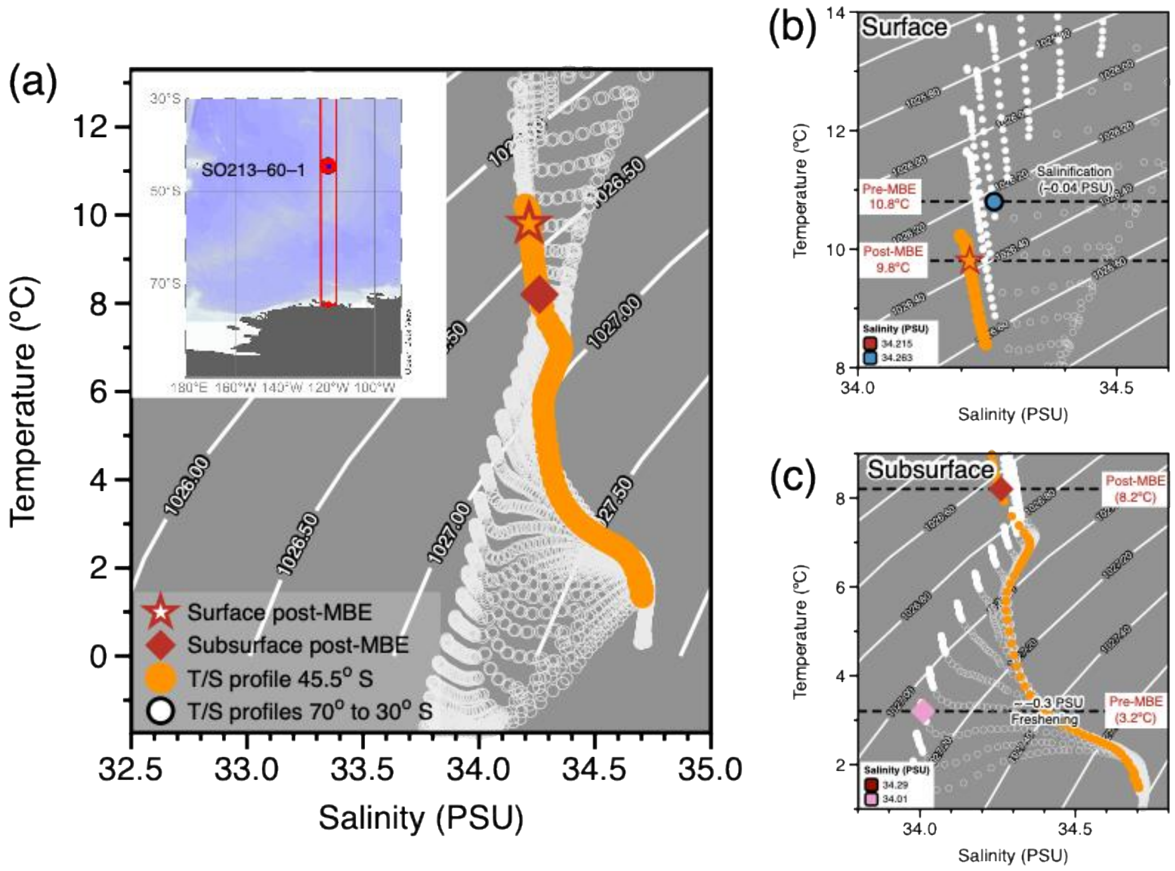

Fig. S5.

Temperature–salinity (T-S) diagram along 119º-120º W (a) Comparison T-S profiles for the transect between 69.5º and 30.5ºS along 119º-120ºW (WOA23) (*75*, *91*), with the 45.5ºS profile highlighted (orange). Empty symbols indicate the spatial variability across latitudes of the T-S profiles. Star and diamond symbols indicate reconstructed post-MBE surface and subsurface temperatures, respectively, with a density contrast of ~–0.3 kg m–3 (table S4). (b) Detail of T-S profiles from 45.5º to 30ºS showing the position of the post-MBE (9.8 °C; star) relative to the 45.5°S profile (orange). The pre-MBE temperature (10.8 °C; blue circle) to extract the corresponding salinity, suggesting a minor salinity increase of +0.01 to +0.05 PSU under a +1 °C warming scenario. (c) Detail of T-S profiles from 69.5º to 45.5º S showing the post-MBE (8.2°C; diamond) relative to the 45.5°S profile (orange). Pre-MBE (3.2 °C) temperatures projected onto 45.5°S and 69.5°S profiles suggest subsurface freshening of ~–0.3 PSU. Filled symbols in (b) and (c) highlight water layer 0–100 (white). Inset in (a) depict the transect used to extract the T-S profiles.

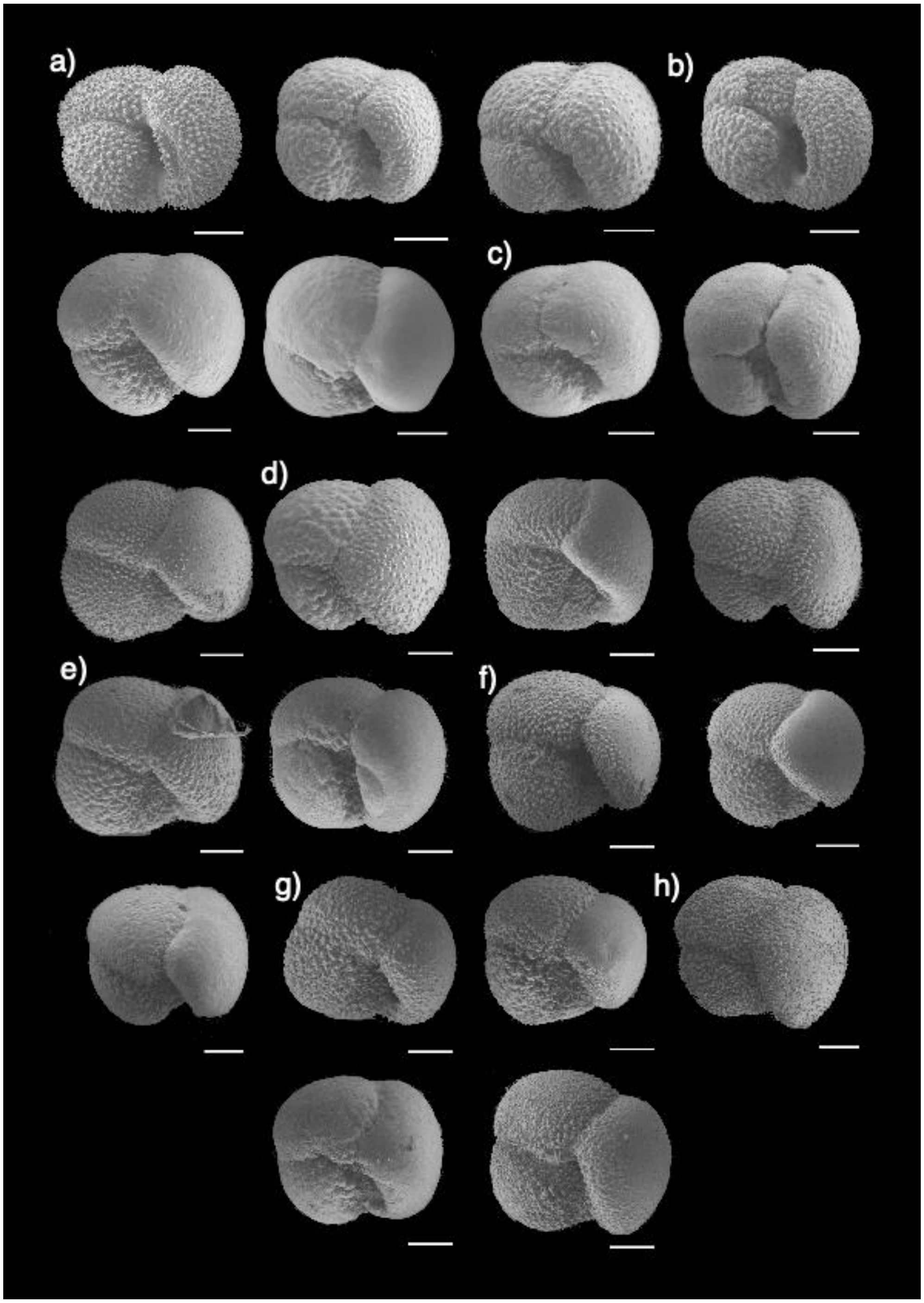

Fig. S6.

Scanning Electronic Microscope (SEM) images showing that *G. inflata* specimens from core SO213–60–1 exhibit medium- to moderately high levels of encrustation. No consistent difference in encrustation levels is observed between pre-MBE samples (a: 4-5 cm; b: 24-25 cm; c: 144-145 cm; d: 176-177 cm; e: 300-301 cm) and post-MBE samples (f: 376-378 cm; g: 408-409 cm; h: 500-501 cm). White lines indicate scale bars (100 μm).

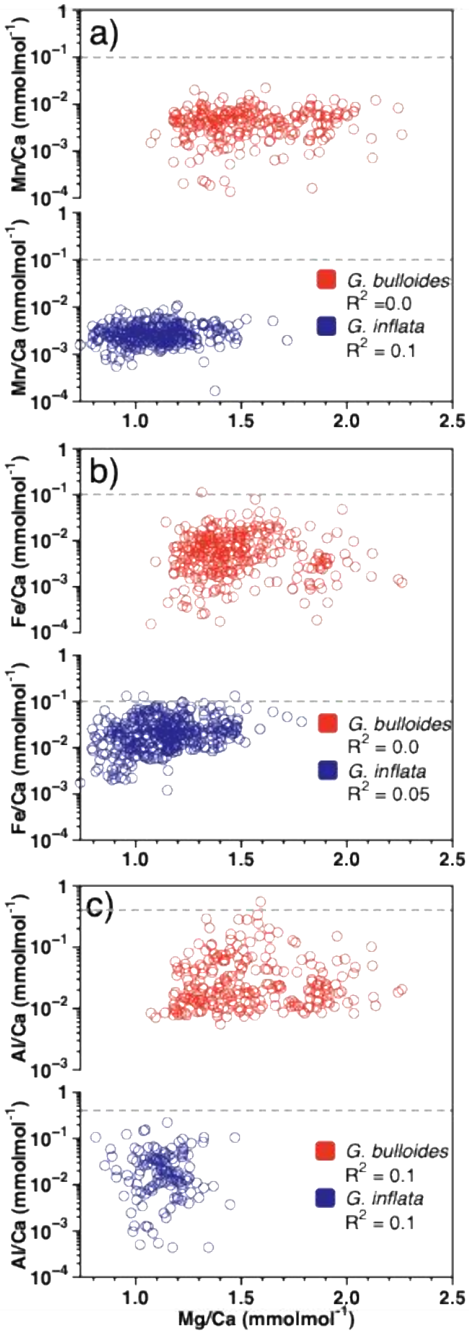

Fig. S7.

Mg/Ca ratios plotted against (a) Mn/Ca, (b) Fe/Ca and (c) Al/Ca for *G. bulloides* (red) and *G. inflata* (blue) from core SO213–60–1. Low elemental ratios and weak correlations ($R^2 < 0.13$) indicate that Mg/Ca values are not affected by contamination. Dashed lines indicate the contamination thresholds: 0.1 mmol mol^{-1} for Mn/Ca and Fe/Ca (*67*), and 0.4 mmol mol^{-1} for Al/Ca (*70*).

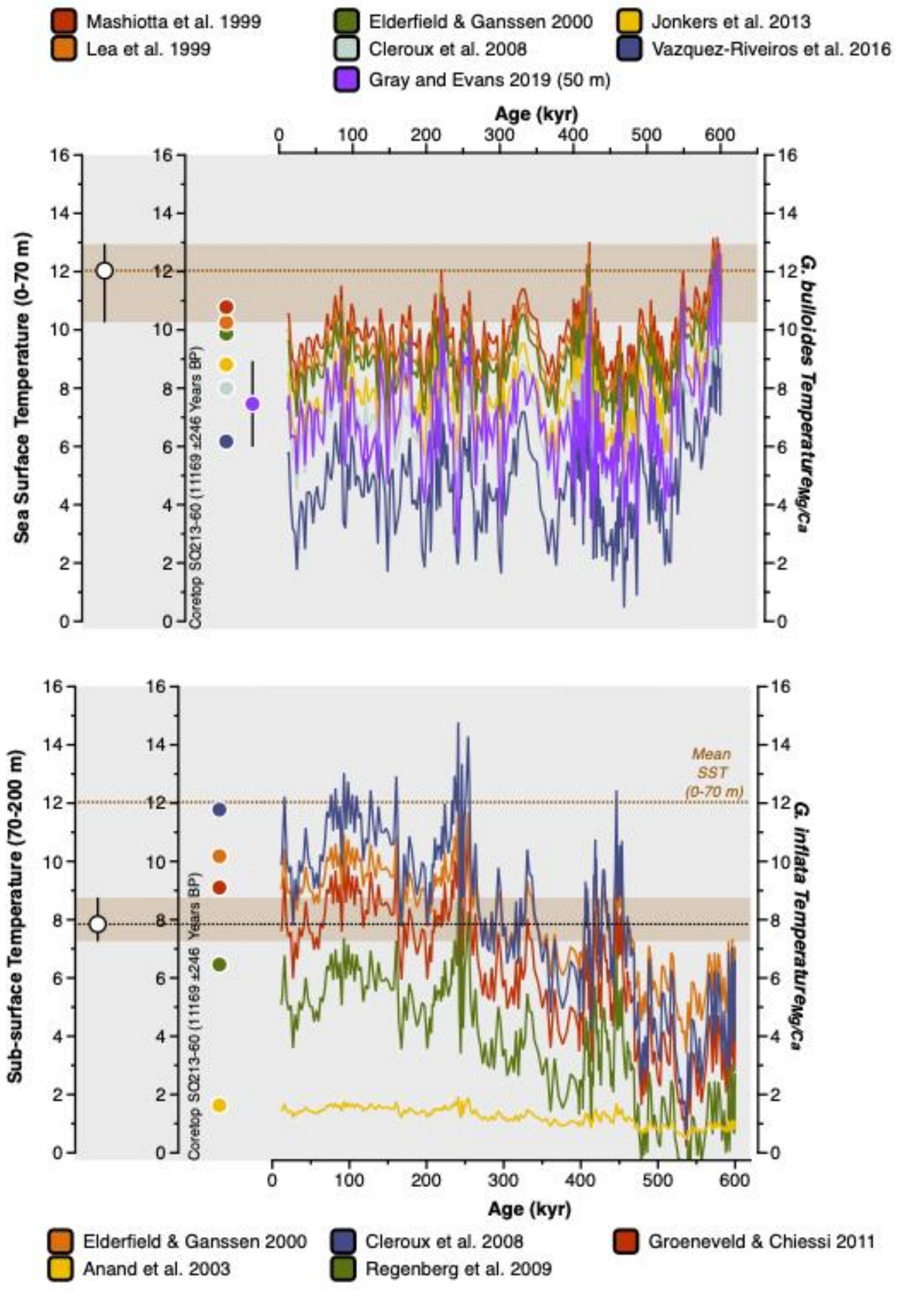

Fig. S8.

Comparison of surface and subsurface Mg/Ca-derived temperatures for *G. bulloides* (top) and *G. inflata* (bottom), calculated using multiple calibrations (see table 3). Red lines indicate the calibration selected for reconstruction and interpretation in the main text. These equations were chosen because the reconstructed core-top temperatures best match austral summer conditions at 0–70 m and 70–200 m depth (white symbols and shaded bands; WOA23) (*75*), consistent with the vertical habitat of each species at similar latitudes in the Atlantic sector of the Southern Ocean (*72*).

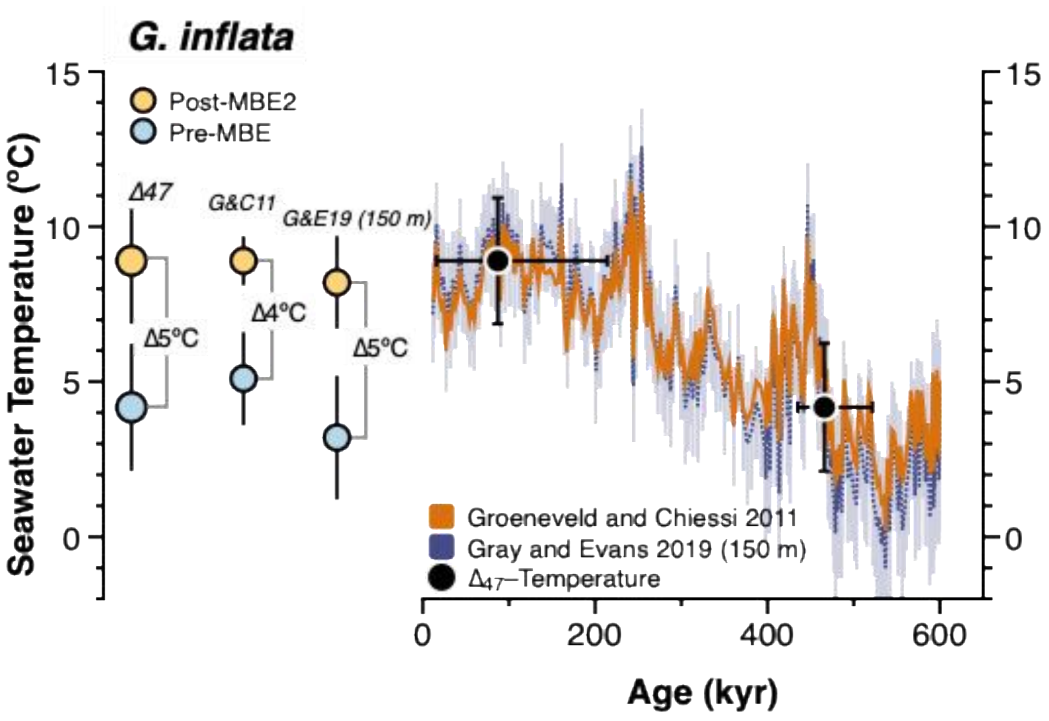

Fig. S9.

Comparison of the thermal gradient (ΔT) across the mid-Brunhes Event (MBE) for *G. inflata*. The left panel shows ΔT estimated derived from Mg/Ca-temperatures calibrations from Groeneveld & Chiessi (*74*) (G&C11) and Gray & Evans (*71*) at 150 m depths (G&E19 150 m), corresponding to the same depths where Δ_{47} thermotry (Δ47) was performed. Error bars represent the error of the mean (2σ) for Δ_{47}, and standard deviation (1σ) for the Mg/Ca-derived temperatures. The right panel shows continuous Mg/Ca temperature reconstructions using both calibrations. Shaded bands indicate the 1σ uncertainty associated with the Gray & Evans equation (*71*). All approaches show consistent temperature trends and comparable thermal gradients within error.

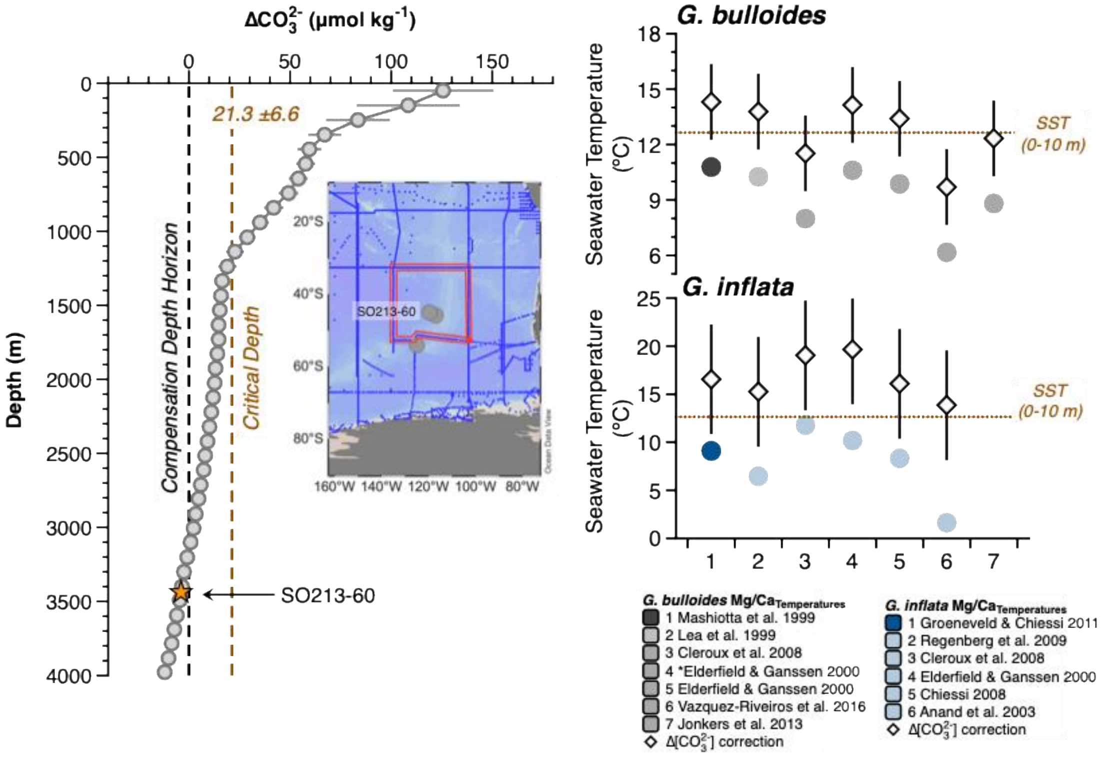

Fig. S10.

Comparison of calcite saturation state and seawater temperature reconstruction from site SO213–60–1. The left panel illustrates depth profile showing the position of the Compensation Depth Horizon (Δ[CO_3^{2-}] = 0 μmol kg^{-1}, where $CaCO_3$ dissolution begins) and the Critical Depth (Δ[CO_3^{2-}] = 21.3 ± 6.6 μmol kg^{-1}, where Mg removal begins) (*76*). The location of core site SO213-60 is indicated. The inset map depicts the transects from the GLODAP database (*94*, *95*) used to calculate Δ[CO_3^{2-}]. The right panel compares the Mg/Ca-derived seawater temperatures for *G. bulloides* and *G. inflata* with modern austral summer sea surface temperatures (0–10 m, dashed lines) at 45–46ºS from WOA18 (*96*). Different Mg/Ca calibrations applied on the core-top SO213–60–1 (this study; see table 3) are shown using filled symbols, while Δ[CO_3^{2-}]-corrected temperatures (empty diamonds) incorporate a dissolution correction of 0.1–0.2°C per μmol kg^{-1} for *G. bulloides* and 0.4–0.6°C per μmol kg^{-1} for *G. inflata* (*76*). Errors bars account for the uncertainty associated with the Critical Depth estimate (*76*). The dissolution correction results in warmer Mg/Ca-derived temperatures, exceeding modern sea surface temperatures at the study site.

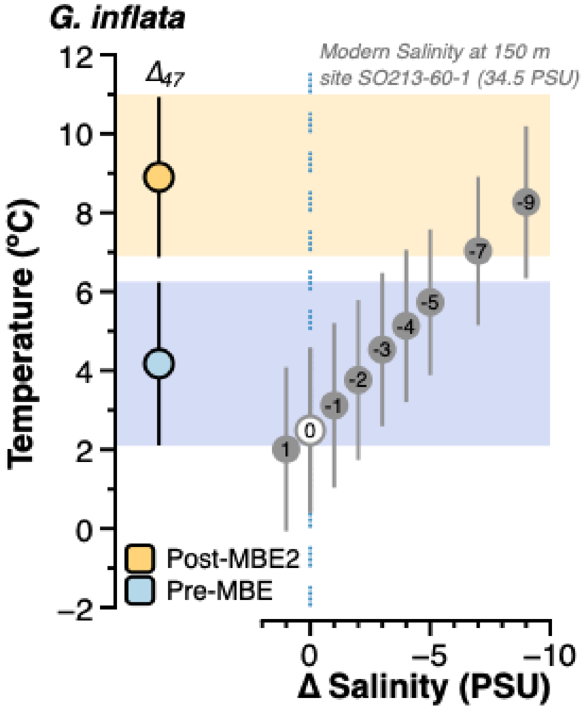

Fig. S11.

Sensitivity of *G. inflata* Mg/Ca-derived temperatures to salinity changes across the Mid-Brunhes Event (MBE). The x-axis represents salinity change (salinity units; PSU) relative to modern conditions (34.5 PSU), while the y-axis shows the corresponding Mg/Ca-derived temperature estimates (°C). Gray circles represent modeled Mg/Ca temperatures under varying salinity conditions, with error bars (1σ) indicating uncertainty (*71*). The blue shaded region represents the Δ_{47}-derived temperature range, providing a salinity-independent benchmark. A salinity decrease of ~7 PSU would be required to push the pre-MBE Mg/Ca-based temperatures beyond Δ_{47} estimates, far exceeding typical glacial–interglacial salinity variability (1–3 PSU) (*97*), indicating that salinity changes have minimal impact on Mg/Ca-based temperature reconstructions.

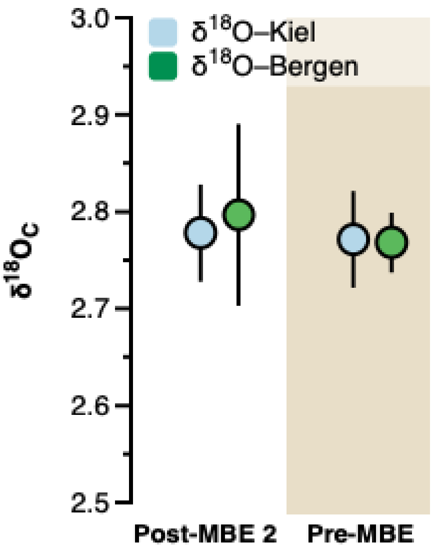

Fig. S12.

Comparison of the $\delta^{18}O$ values measured at the same sampling depths (see table S2) by the Kiel and Bergen laboratories. Results show agreement within analytical error. Error bars represent the analytical standard errors (1σ) for Kiel laboratory and the standard error of the mean (2σ) for the Bergen laboratory.

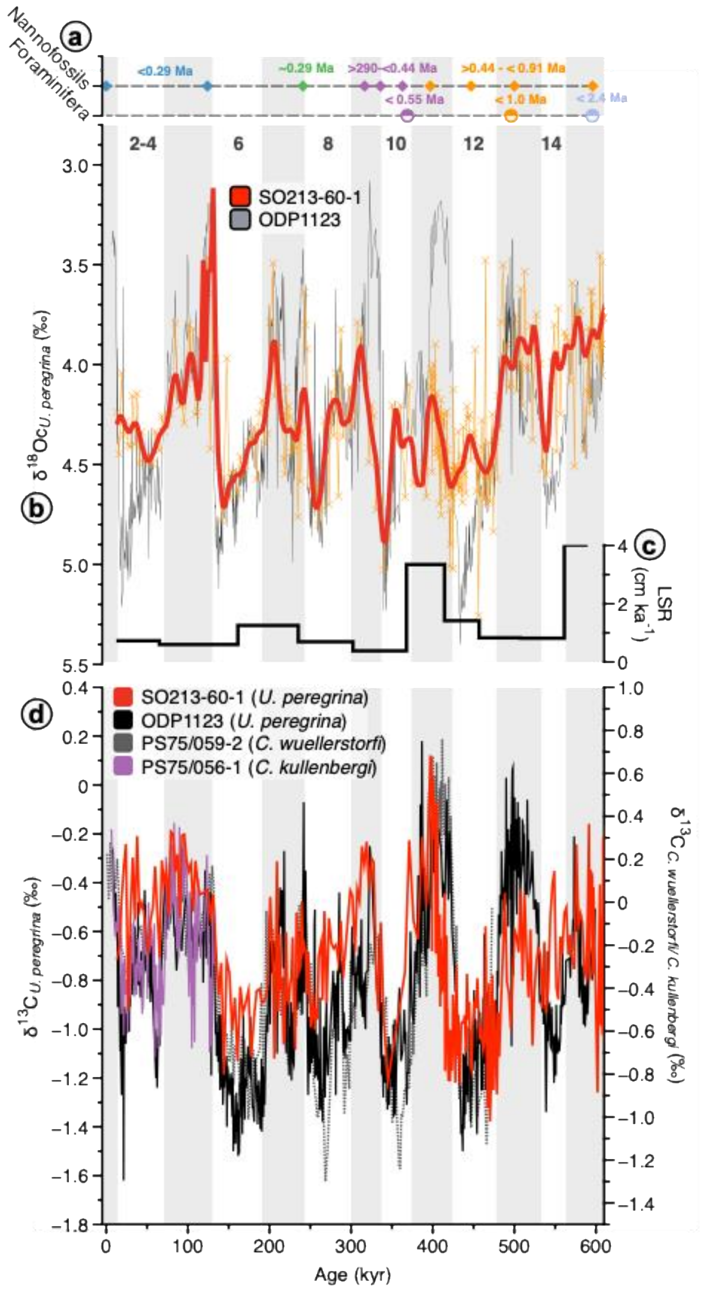

Fig. S13.

(a) Nannofossil and foraminiferal biodatums (*61*) were used to support the (b) correlation of benthic $\delta^{18}O_{U.\ peregrina}$ record from core SO213–60–1 (red) and that from core ODP 1123 (gray) (*83*). The thick red line in panel b depicts Loess smoothing of the $\delta^{18}O$ data from core SO213–60–1. (c) Linear sedimentation rates of core SO213–60–1. (d) The robustness of the age model is further validated by a good match in the overall trend and timing of the MIS 11 peak in the benthic $\delta^{13}C$ records with three regional cores: SO213–60–1 (red), ODP 1123 (gray) (*83*) and the independently-dated PS75/059–2 (black) and PS75/056–1 (purple) (*86*).

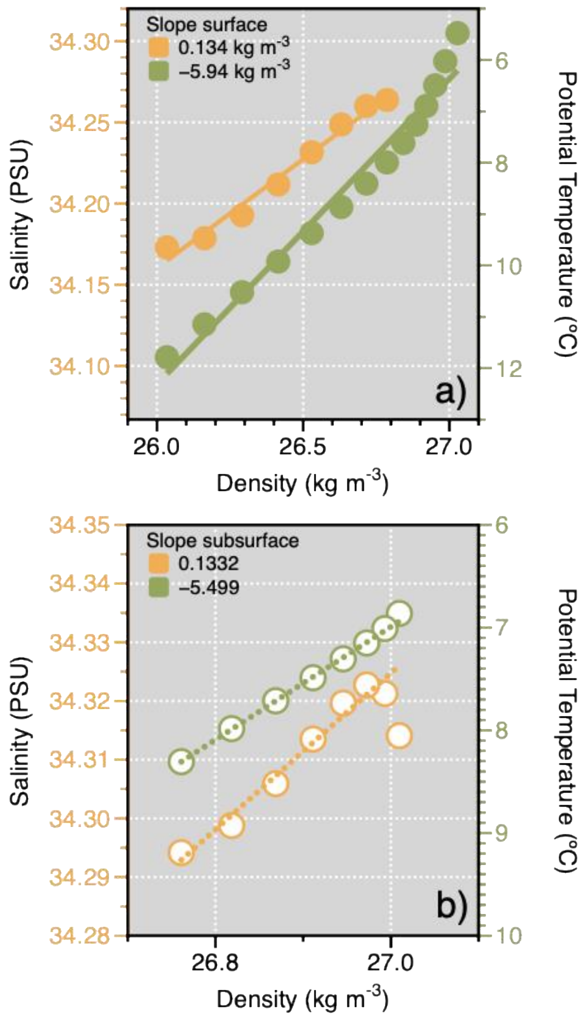

Fig. S14.

Relationship between potential density, temperature, and salinity along the 50–42°S transect (WOA23) (*75*, *91*). (a) Surface ocean: temperature (green) and salinity (orange) plotted against potential density, showing contrasting sensitivities. Temperature decreases sharply with increasing density (slope = −4 °C kg^{-1} m^3), while salinity shows a modest increase (slope = 0.12 PSU kg^{-1} m^3). (b) Subsurface ocean: similar relationships are observed, with temperature again exhibiting a strong inverse correlation (slope = −5 °C kg^{-1} m^3) and salinity a weaker positive trend (slope = 0.2 PSU kg^{-1} m^3). These patterns indicate that stratification is primarily controlled by temperature at both depths.

Table S1.

Summary of Δ_{47} results for the selected depths across the Mid-Brunhes Event (MBE); pre-MBE (16–214 ka) and post-MBE 2 (423–510 ka) time windows. The average Mg/Ca temperatures from depth intervals considered for the Δ_{47} analysis are presented for comparison.

Depth (cm)	Age (ka)	Replicates (#)	Median Temperature Δ_{47} (ºC)	Error of the mean (2σ) (ºC)	Mg/Ca Average Temperature (ºC)
**4-5**	16	14	8.9	±2.0	8.9±0.8
**56-57**	87	9			
**144-145**	214	14			
**376-377**	423	13	4.2	±2.1	5.1±1.5
**408-409**	433	11			
**500-501**	510	10			

Table S2.

To assess whether *G. bulloides* and *G. inflata* record distinct water masses in the South Pacific, we conducted independent samples t-tests comparing their Mg/Ca-derived temperature ranges over the last 200 kyr. Our hypothesis was that if each species records a different water mass, their temperature distributions should be significantly different. Welch's t-tests yielded statistically significant differences ($p < 0.001$) between $SST_{Mg/Ca}$ (*G. bulloides*) and $SubT_{Mg/Ca}$ (*G. inflata*), rejecting the null hypothesis and indicating that the two species capture distinct water masses at each latitude (*G. bulloides* = SST = SAMW and *G. inflata* = SubT = AAIW; see Fig. 1, B and C). To further test whether these water masses remain distinct across latitudes, we compared SSTs at 54ºS (PS75/059–2) with SubTs at 45ºS (SO213–60–1). Welch's t-test found no statistically significant difference between these groups, supporting the hypothesis that both proxies record the same water mass (AAIW) but at different latitudes. Statistical tests were performed using software Jamovi (*98*).

Core	Welch's t	df	p	Alternative Hypothesis (H_a)
SO213–59–2	16.134	145.36	<0.001	$\mu SST_{Mg/Ca} \neq \mu SubT_{Mg/Ca}$
PS75/059–2	16.173	135.55	<0.001	$\mu SST_{Mg/Ca} \neq \mu SubT_{Mg/Ca}$
SO213–60–1	-10.694	118.52	<0.001	$\mu SST_{Mg/Ca} \neq \mu SubT_{Mg/Ca}$
PS75/059–2 vs. SO213–60–1	-0.61299	116.03	0.541	$\mu SST(54ºS)_{Mg/Ca} \neq \mu SubT(45ºS)_{Mg/Ca}$

Table S3.

Estimated salinity changes derived from observed $\delta^{18}O_{SW}$ shifts using regional $\delta^{18}O_{SW}$ −salinity relationships for the Southern Ocean ($\delta^{18}O_{SW} = 0.24\ x\ Salinity - 8.45$) and Subtropical Pacific ($\delta^{18}O_{SW} = 0.45\ x\ Salinity - 15.29$) (*26*).

**Water depth**	$\boldsymbol{\Delta\delta^{18}O_{SW}}$ **(‰)**	**Southern Ocean** $\boldsymbol{0.24\ x\ Salinity}$ **(Salinity change, PSU)**	**Subtropical Pacific** $\boldsymbol{0.45\ x\ Salinity}$ **(Salinity change, PSU)**
**Surface**	0.1	0.417	0.22
**Subsurface**	1.56	6.5	3.47

Table S4.

Surface–subsurface potential density differences (Δρ) (*92*) under varying temperature and salinity scenarios simulating MBE conditions (IES80). Surface densities incorporate warming and salinity increases; subsurface values simulate cooling with progressive freshening.

**Surface Condition**	**Temp (°C)**	**Sal (PSU)**	**Density (kg/m³)**	**Subsurface Condition**	**Temp (°C)**	**Sal (PSU)**	**Density (kg/m³)**	**ΔDensity (kg/m³)**
**Post-MBE**								
Reference	9.8	34.18	1026.346	Reference	8.2	34.21	1026.624	–0.278
		34.25	1026.400			34.31	1026.704	–0.304
**Pre-MBE**								
Warming + 34.25 (+0.05PSU)	10.8	34.3	1026.267	Cooling + 34.21 (–0.3PSU)	3.2	33.91	1026.997	–0.73
				Cooling + 34.21 (–0.5PSU)	3.2	33.71	1026.837	–0.570
				Cooling + 34.21 (–1.21PSU)	3.2	33.00	1026.271	–0.004

Table S5.

Summary of Mg/Ca temperature equations assessed for the reconstruction of water temperatures.
* Multispecies calibration.

	**Mg/Ca = B exp(AT)**	
G. bulloides	**B**	**A**
[1] Mashiotta et al. (*73*)	0.474	0.107
[2] Lea et al. (*99*)	0.528	0.102
[3] Elderfield and Ganssen (*100*)	0.52	0.1
[4] Cleroux et al. (*101*)	0.78	0.82
[5] Jonkers et al. (*102*)	0.68	0.09
[6] Vazques-Riveiros et al. (*103*)	1.006	0.065
[7] Gray and Evans (*71*)	Mg/Ca = exp(0.036×(S-35) + 0.061×T + -0.88×(pH-8) + 0.21)	
G. inflata		
[8] Elderfield and Ganssen (*100*)*	0.52	0.1
[9] Anand et al. (*104*)	0.56	0.58
[10] Cleroux et al. (*101*)	0.71	0.056
[11] Regenberg et al. (*105*)*	0.842	0.083
[12] Groeneveld and Chiessi (*74*)	0.72	0.76
[13] Gray and Evans (*71*)*	Mg/Ca = exp(0.036×(S-35) + 0.061×T + -0.73×(pH-8))	

Table S6.

Summary of the salinity, alkalinity and ΔpCO_2 values (*96*, *97*) used for the Mg/Ca temperature estimates of core SO213–60–1 through the MgCaRB online tool.

	Salinity (PSU)	Alkalinity (µmol Kg^{-1})	ΔpCO_2 (µAtm)
G. bulloides			
50 m	34.36	2297	-55.84
G. inflata			
150 m	34.52	2300.4	-0.75

Data S1. (separate file)

Data from core SO213–60–1 and multicore SO213–60–2 including oxygen isotopes, trace element ratios (Mg/Ca, Al/Ca, Fe/Ca and Mn/Ca), and Mg/Ca derived temperature estimates for *G. bulloides* and *G. inflata*, as well as clumped isotopes, clumped isotope derived temperature estimates for *G. inflata.*

REFERENCES

1. D. M. Sigman, F. Fripiat, A. S. Studer, P. C. Kemeny, A. Martínez-García, M. P. Hain, X. Ai, X. Wang, H. Ren, G. H. Haug, The Southern Ocean during the ice ages: A review of the Antarctic surface isolation hypothesis, with comparison to the North Pacific. *Quat. Sci. Rev.* **254**, 106732 (2021).

2. T. DeVries, The ocean carbon cycle. *Annu. Rev. Env. Resour.* **47**, 317–341 (2022).

3. I. Marinov, A. Gnanadesikan, J. R. Toggweiler, J. L. Sarmiento, The Southern Ocean biogeochemical divide. *Nature* **441**, 964–967 (2006).

4. V. Pellichero, J.-B. Sallée, C. C. Chapman, S. M. Downes, The southern ocean meridional overturning in the sea-ice sector is driven by freshwater fluxes. *Nat. Commun.* **9**, 1789 (2018).

5. C. L. Sabine, R. A. Feely, N. Gruber, R. M. Key, K. Lee, J. L. Bullister, R. Wanninkhof, C. S. Wong, D. W. R. Wallace, B. Tilbrook, F. J. Millero, T.-H. Peng, A. Kozyr, T. Ono, A. F. Rios, The oceanic sink for anthropogenic CO_2. *Science* **305**, 367–371 (2004).

6. N. Gruber, P. Landschützer, N. S. Lovenduski, The variable southern ocean carbon sink. *Ann. Rev. Mar. Sci.* **11**, 159–186 (2019).

7. A. Martínez-Garcia, A. Rosell-Melé, S. L. Jaccard, W. Geibert, D. M. Sigman, G. H. Haug, Southern Ocean dust-climate coupling over the past four million years. *Nature* **476**, 312–315 (2011).

8. P. R. Oke, M. H. England, Oceanic response to changes in the latitude of the southern hemisphere subpolar westerly winds. *J. Climate* **17**, 1040–1054 (2004).

9. W. P. Sijp, M. H. England, The effect of a northward shift in the southern hemisphere westerlies on the global ocean. *Prog. Oceanogr.* **79**, 1–19 (2008).

10. J. Ribbe, Intermediate water mass production controlled by southern hemisphere winds. *Geophys. Res. Lett.* **28**, 535–538 (2001).

11. S. R. Rintoul, C. W. Hughes, D. Olbers, “The Antarctic Circumpolar Current System,” in *Ocean Circulation and Climate - Observing and Modelling the Global Ocean* (Elsevier, 2001), vol. 77, pp. 271–302.

12. F. A. Haumann, N. Gruber, M. Münnich, I. Frenger, S. Kern, Sea-ice transport driving Southern Ocean salinity and its recent trends. *Nature* **537**, 89–92 (2016).

13. H. C. Bostock, P. J. Sutton, M. J. M. Williams, B. N. Opdyke, Reviewing the circulation and mixing of Antarctic Intermediate Water in the South Pacific using evidence from geochemical tracers and Argo float trajectories. *Deep-Sea Res. I Oceanogr. Res. Pap.* **73**, 84–98 (2013).

14. K. Hanawa, L. D. Talley, “Mode waters,” in *Ocean Circulation and Climate - Observing and Modelling the Global Ocean* (Elsevier, 2001), vol. 77, pp. 373–386.

15. N. V. Zilberman, D. H. Roemmich, S. T. Gille, Meridional volume transport in the South Pacific: Mean and SAM-related variability. *J. Geophys. Res. Oceans* **119**, 2658–2678 (2014).

16. R. François, M. A. Altabet, E.-F. Yu, D. M. Sigman, M. P. Bacon, M. Frank, G. Bohrmann, G. Bareille, L. D. Labeyrie, Contribution of Southern Ocean surface-water stratification to low atmospheric CO_2 concentrations during the last glacial period. *Nature* **389**, 929–935 (1997).

17. D. Lüthi, M. Le Floch, B. Bereiter, T. Blunier, J.-M. Barnola, U. Siegenthaler, D. Raynaud, J. Jouzel, H. Fischer, K. Kawamura, T. F. Stocker, High-resolution carbon dioxide concentration record 650,000-800,000 years before present. *Nature* **453**, 379–382 (2008).

18. B. Bereiter, S. Eggleston, J. Schmitt, C. Nehrbass-Ahles, T. F. Stocker, H. Fischer, S. Kipfstuhl, J. Chappellaz, Revision of the EPICA Dome C CO_2 record from 800 to 600 kyr before present. *Geophys. Res. Lett.* **42**, 542–549 (2015).

19. A. E. S. Kemp, I. Grigorov, R. B. Pearce, A. C. N. Garabato, Migration of the Antarctic Polar Front through the mid-Pleistocene transition: Evidence and climatic implications. *Quat. Sci. Rev.* **29**, 1993–2009 (2010).

20. Q. Yin, Insolation-induced mid-Brunhes transition in Southern Ocean ventilation and deep-ocean temperature. *Nature* **494**, 222–225 (2013).

21. A. M. Barth, P. U. Clark, N. S. Bill, F. He, N. G. Pisias, Climate evolution across the Mid-Brunhes transition. *Climate Past Discuss.* **14**, 2071–2087 (2018).

22. T. Mitsui, N. Boers, Machine learning approach reveals strong link between obliquity amplitude increase and the Mid-Brunhes transition. *Quat. Sci. Rev.* **277**, 107344 (2022).

23. N. Bouttes, N. Vazquez Riveiros, A. Govin, D. Swingedouw, M. F. Sanchez-Goni, X. Crosta, D. M. Roche, Carbon 13 isotopes reveal limited ocean circulation changes between interglacials of the last 800 ka. *Paleoceanogr. Paleoclimatol.* **35**, e2019PA003776 (2020).

24. N. Bouttes, D. Swingedouw, D. M. Roche, M. F. Sanchez-Goni, X. Crosta, Response of the carbon cycle in an intermediate complexity model to the different climate configurations of the last nine interglacials. *Clim. Past* **14**, 239–253 (2018).

25. R. Tapia, D. Nürnberg, S. L. Ho, F. Lamy, J. Ullermann, R. Gersonde, R. Tiedemann, Glacial differences of Southern Ocean Intermediate Waters in the Central South Pacific. *Quat. Sci. Rev.* **208**, 105–117 (2019).

26. R. Tapia, D. Nürnberg, T. Ronge, R. Tiedemann, Disparities in glacial advection of Southern Ocean Intermediate Water to the South Pacific Gyre. *Earth Planet. Sci. Lett.* **410**, 152–164 (2015).

27. Z. Li, S. Groeskamp, I. Cerovečki, M. H. England, The origin and fate of antarctic intermediate water in the southern ocean. *J. Phys. Oceanogr.* **52**, 2873–2890 (2022).

28. J. M. Muratli, Z. Chase, A. C. Mix, J. McManus, Increased glacial-age ventilation of the Chilean margin by Antarctic Intermediate Water. *Nat. Geosci.* **3**, 23–26 (2010).

29. G. Martínez-Méndez, D. Hebbeln, M. Mohtadi, F. Lamy, R. De Pol-Holz, D. Reyes-Macaya, T. Freudenthal, Changes in the advection of Antarctic Intermediate Water to the northern Chilean coast during the last 970 kyr. *Paleoceanography* **28**, 607–618 (2013).

30. L. D. Pena, I. Cacho, P. Ferretti, M. A. Hall, El Niño-Southern Oscillation-like variability during glacial terminations and interlatitudinal teleconnections. *Paleoceanography* **23**, PA3101 (2008).

31. F. Lamy, R. Gersonde, G. Winckler, O. Esper, A. Jaeschke, G. Kuhn, J. Ullermann, A. Martinez-Garcia, F. Lambert, R. Kilian, Increased dust deposition in the Pacific Southern Ocean during glacial periods. *Science* **343**, 403–407 (2014).

32. R. P. Abernathey, I. Cerovecki, P. R. Holland, E. Newsom, M. Mazloff, L. D. Talley, Water-mass transformation by sea ice in the upper branch of the Southern Ocean overturning. *Nat. Geosci.* **9**, 596–601 (2016).

33. L. Li, Z. Liu, C. Zhu, C. He, B. Otto-Bliesner, Shallowing glacial antarctic intermediate water by changes in sea ice and hydrological cycle. *Geophys. Res. Lett.* **48**, e2021GL094317 (2021).

34. T. A. Ronge, S. Steph, R. Tiedemann, M. Prange, U. Merkel, D. Nürnberg, G. Kuhn, Pushing the boundaries: Glacial/interglacial variability of intermediate and deep waters in the southwest Pacific over the last 350,000 years. *Paleoceanography* **30**, 23–38 (2015).

35. J. Jones, K. E. Kohfeld, H. Bostock, X. Crosta, M. Liston, G. Dunbar, Z. Chase, A. Leventer, H. Anderson, G. Jacobsen, Sea ice changes in the southwest Pacific sector of the Southern Ocean during the last 140,000 years. *Clim. Past* **18**, 465–483 (2022).

36. E. W. Wolff, H. Fischer, F. Fundel, U. Ruth, B. Twarloh, G. C. Littot, R. Mulvaney, R. Röthlisberger, M. de Angelis, C. F. Boutron, M. Hansson, U. Jonsell, M. A. Hutterli, F. Lambert, P. Kaufmann, B. Stauffer, T. F. Stocker, J. P. Steffensen, M. Bigler, M. L. Siggaard-Andersen, R. Udisti, S. Becagli, E. Castellano, M. Severi, D. Wagenbach, C. Barbante, P. Gabrielli, V. Gaspari, Southern Ocean sea-ice extent, productivity and iron flux over the past eight glacial cycles. *Nature* **440**, 491–496 (2006).

37. B. J. Davison, A. E. Hogg, N. Gourmelen, L. Jakob, J. Wuite, T. Nagler, C. A. Greene, J. Andreasen, M. E. Engdahl, Annual mass budget of Antarctic ice shelves from 1997 to 2021. *Sci. Adv.* **9**, eadi0186 (2023).

38. T. Rackow, C. Wesche, R. Timmermann, H. H. Hellmer, S. Juricke, T. Jung, A simulation of small to giant Antarctic iceberg evolution: Differential impact on climatology estimates. *J. Geophys. Res. Oceans* **122**, 3170–3190 (2017).

39. H. D. Pritchard, S. R. M. Ligtenberg, H. A. Fricker, D. G. Vaughan, M. R. van den Broeke, L. Padman, Antarctic ice-sheet loss driven by basal melting of ice shelves. *Nature* **484**, 502–505 (2012).

40. M. A. Depoorter, J. L. Bamber, J. A. Griggs, J. T. M. Lenaerts, S. R. M. Ligtenberg, M. R. van den Broeke, G. Moholdt, Calving fluxes and basal melt rates of Antarctic ice shelves. *Nature* **502**, 89–92 (2013).

41. J. Wang, Z. Tang, D. J. Wilson, F. Chang, Z. Xiong, D. Li, T. Li, Ocean-forced instability of the west antarctic ice sheet since the mid-pleistocene. *Geochem. Geophys. Geosyst.* **23**, e2022GC010470 (2022).

42. L. Jebasinski, D. A. Frick, A. K. I. U. Kapuge, C. Basak, M. Saavedra-Pellitero, G. Winckler, F. Lamy, J. Gottschalk, Southern Ocean evidence for recurring West Antarctic Ice Sheet destabilization during Marine Isotope Stage 11. *Nat. Commun.* **16**, 9138 (2025).

43. D. J. Wilson, R. A. Bertram, E. F. Needham, T. van de Flierdt, K. J. Welsh, R. M. McKay, A. Mazumder, C. R. Riesselman, F. J. Jimenez-Espejo, C. Escutia, Ice loss from the East Antarctic Ice Sheet during late Pleistocene interglacials. *Nature* **561**, 383–386 (2018).

44. R. B. Alley, S. Anandakrishnan, T. K. Dupont, B. R. Parizek, D. Pollard, Effect of sedimentation on ice-sheet grounding-line stability. *Science* **315**, 1838–1841 (2007).

45. N. Merino, J. Le Sommer, G. Durand, N. C. Jourdain, G. Madec, P. Mathiot, J. Tournadre, Antarctic icebergs melt over the Southern Ocean: Climatology and impact on sea ice. *Ocean Model.* **104**, 99–110 (2016).

46. F. Lamy, G. Winckler, H. W. Arz, J. R. Farmer, J. Gottschalk, L. Lembke-Jene, J. L. Middleton, M. van der Does, R. Tiedemann, C. Alvarez Zarikian, C. Basak, A. Brombacher, L. Dumm, O. M. Esper, L. C. Herbert, S. Iwasaki, G. Kreps, V. J. Lawson, L. Lo, E. Malinverno, A. Martinez-

Garcia, E. Michel, S. Moretti, C. M. Moy, A. C. Ravelo, C. R. Riesselman, M. Saavedra-Pellitero, H. Sadatzki, I. Seo, R. K. Singh, R. A. Smith, A. L. Souza, J. S. Stoner, M. Toyos, I. M. V. P. de Oliveira, S. Wan, S. Wu, X. Zhao, Five million years of Antarctic Circumpolar Current strength variability. *Nature* **627**, 789–796 (2024).

47. C. D. Hillenbrand, G. Kuhn, T. Frederichs, Record of a Mid-Pleistocene depositional anomaly in West Antarctic continental margin sediments: An indicator for ice-sheet collapse? *Quat. Sci. Rev.* **28**, 1147–1159 (2009).

48. R. F. Weiss, Carbon dioxide in water and seawater: The solubility of a non-ideal gas. *Mar. Chem.* **2**, 203–215 (1974).

49. T. Takahashi, J. Olafsson, J. G. Goddard, D. W. Chipman, S. C. Sutherland, Seasonal variation of CO_2 and nutrients in the high-latitude surface oceans: A comparative study. *Global Biogeochem. Cycles* **7**, 843–878 (1993).

50. P. Atkins, J. de Paula, J. Keeler, *Atkins' Physical Chemistry* (Oxford Univ. Press, 2022).

51. S. L. Ho, G. Mollenhauer, F. Lamy, A. Martínez-Garcia, M. Mohtadi, R. Gersonde, D. Hebbeln, S. Nunez-Ricardo, A. Rosell-Melé, R. Tiedemann, Sea surface temperature variability in the Pacific sector of the Southern Ocean over the past 700 kyr. *Paleoceanography* **27**, PA4202 (2012).

52. M. Duffy, C. R. Riesselman, O. Esper, V. Sinnen, L. Lembke-Jene, C. M. Moy, F. Lamy, Northward shift of south pacific polar frontal system across the mid-pleistocene transition: A 1.4 myr continuous diatom paleoenvironmental record from the pacific sector of the antarctic circumpolar current. *Paleoceanogr. Paleoclimatol.* **40**, e2025PA005171 (2025).

53. A. N. LeGrande, G. A. Schmidt, Global gridded data set of the oxygen isotopic composition in seawater. *Geophys. Res. Lett.* **33**, L12604 (2006).

54. M. Crundwell, G. Scott, T. Naish, L. Carter, Glacial–interglacial ocean climate variability from planktonic foraminifera during the Mid-Pleistocene transition in the temperate Southwest Pacific, ODP Site 1123. *Palaeogeogr. Palaeoclimatol. Palaeoecol.* **260**, 202–229 (2008).

55. M. W. Wara, A. C. Ravelo, M. L. Delaney, Permanent El Niño-like conditions during the Pliocene warm period. *Science* **309**, 758–761 (2005).

56. D. M. Sigman, M. P. Hain, G. H. Haug, The polar ocean and glacial cycles in atmospheric CO_2 concentration. *Nature* **466**, 47–55 (2010).

57. A. Martínez-Garcia, A. Rosell-Melé, W. Geibert, R. Gersonde, P. Masqué, V. Gaspari, C. Barbante, Links between iron supply, marine productivity, sea surface temperature, and CO_2 over the last 1.1 Ma. *Paleoceanography* **24**, PA1207 (2009).

58. Q. Li, M. H. England, A. M. Hogg, S. R. Rintoul, A. K. Morrison, Abyssal ocean overturning slowdown and warming driven by Antarctic meltwater. *Nature* **615**, 841–847 (2023).

59. L. Yi, M. Medina-Elizalde, L. Tan, D. B. Kemp, Y. Li, G. Kletetschka, Q. Xie, H. Yao, H. He, C. Deng, J. G. Ogg, Plio-Pleistocene deep-sea ventilation in the eastern Pacific and potential linkages with Northern Hemisphere glaciation. *Sci. Adv.* **9**, eadd1467 (2023).

60. H. Huang, J. Fietzke, M. Gutjahr, M. Frank, G. Kuhn, X. Zhang, C.-D. Hillenbrand, D. Li, J. Hu, J. Yu, Enhanced deep Southern Ocean stratification during the lukewarm interglacials. *Nat. Commun.* **16**, 8856 (2025).

61. R. Tiedemann, F. Lamy, Participants, “FS Sonne Fahrtbericht/Cruise Report SO213 - SOPATRA: South Pacific Paleoceanographic Transects - Geodynamic and Climatic Variability in Space and Time, Leg 1: Valparaiso/Chile - Valparaiso/Chile, 27.012.2010 - 12.01.2011, Leg 2: Valparaiso/Chile - Wellington/New Zealand, 12.01.2011 - 07.03.2011” (Alfred Wegener Institute, Helmholtz Center for Polar and Marine Research, 2014).

62. M. Molina-Kescher, M. Frank, E. Hathorne, South Pacific dissolved Nd isotope compositions and rare earth element distributions: Water mass mixing versus biogeochemical cycling. *Geochim. Cosmochim. Acta* **127**, 171–189 (2014).

63. L. Jonkers, A. Gopalakrishnan, L. Weßel, C. M. Chiessi, J. Groeneveld, P. Monien, D. Lessa, R. Morard, Morphotype and crust effects on the geochemistry of *Globorotalia inflata*. *Paleoceanogr. Paleoclimatol.* **36**, e2021PA004224 (2021).

64. N. Meinicke, M. A. Reimi, A. C. Ravelo, A. N. Meckler, Coupled Mg/Ca and clumped isotope measurements indicate lack of substantial mixed layer cooling in the Western Pacific Warm Pool during the last ~5 million years. *Paleoceanogr. Paleoclimatol.* **36**, e2020PA004115 (2021).

65. S. M. Bernasconi, M. Daëron, K. D. Bergmann, M. Bonifacie, A. N. Meckler, H. P. Affek, N. Anderson, D. Bajnai, E. Barkan, E. Beverly, D. Blamart, L. Burgener, D. Calmels, C. Chaduteau, M. Clog, B. Davidheiser-Kroll, A. Davies, F. Dux, J. Eiler, B. Elliott, A. C. Fetrow, J. Fiebig, S. Goldberg, M. Hermoso, K. W. Huntington, E. Hyland, M. Ingalls, M. Jaggi, C. M. John, A. B. Jost, S. Katz, J. Kelson, T. Kluge, I. J. Kocken, A. Laskar, T. J. Leutert, D. Liang, J. Lucarelli, T. J. Mackey, X. Mangenot, N. Meinicke, S. E. Modestou, I. A. Müller, S. Murray, A. Neary, N. Packard, B. H. Passey, E. Pelletier, S. Petersen, A. Piasecki, A. Schauer, K. E. Snell, P. K. Swart, A. Tripati, D. Upadhyay, T. Vennemann, I. Winkelstern, D. Yarian, N. Yoshida, N. Zhang, M. Ziegler, Intercarb: A community effort to improve interlaboratory standardization of the carbonate clumped isotope thermometer using carbonate standards. *Geochem. Geophys. Geosyst.* **22**, e2020GC009588 (2021).

66. N. Meinicke, S. L. Ho, B. Hannisdal, D. Nürnberg, A. Tripati, R. Schiebel, A. N. Meckler, A robust calibration of the clumped isotopes to temperature relationship for foraminifers. *Geochim. Cosmochim. Acta* **270**, 160–183 (2020).

67. S. Barker, M. Greaves, H. Elderfield, A study of cleaning procedures used for foraminiferal Mg/Ca paleothermometry. *Geochem. Geophys. Geosyst.* **4**, 8407 (2003).

68. R. Tapia, "Paleo-water column structure in the South Pacific: Evidence from foraminiferal $\delta^{18}O$ and Mg/Ca," thesis, Christian-Albrechts-Universität zu Kiel (2016).

69. M. Greaves, N. Caillon, H. Rebaubier, G. Bartoli, S. Bohaty, I. Cacho, L. Clarke, M. Cooper, C. Daunt, M. Delaney, P. deMenocal, A. Dutton, S. Eggins, H. Elderfield, D. Garbe-Schoenberg, E. Goddard, D. Green, J. Groeneveld, D. Hastings, E. Hathorne, K. Kimoto, G. Klinkhammer, L. Labeyrie, D. W. Lea, T. Marchitto, M. A. Martínez-Botí, P. G. Mortyn, Y. Ni, D. Nuernberg, G. Paradis, L. Pena, T. Quinn, Y. Rosenthal, A. Russell, T. Sagawa, S. Sosdian, L. Stott, K. Tachikawa, E. Tappa, R. Thunell, P. A. Wilson, Interlaboratory comparison study of calibration standards for foraminiferal Mg/Ca thermometry. *Geochem. Geophys. Geosyst.* **9**, Q08010 (2008).

70. M. Greaves, "Trace elements in marine biogenic carbonates: Analysis and application to past ocean chemistry," thesis, School of Ocean and Earth Science, University of Southampton (2008).

71. W. R. Gray, D. Evans, Nonthermal influences on mg/ca in planktonic foraminifera: A review of culture studies and application to the last glacial maximum. *Paleoceanogr. Paleoclimatol.* **34**, 306–315 (2019).

72. P. G. Mortyn, C. D. Charles, Planktonic foraminiferal depth habitat and δ^{18}O calibrations: Plankton tow results from the Atlantic sector of the Southern Ocean. *Paleoceanography* **18**, 1037 (2003).

73. T. A. Mashiotta, D. W. Lea, H. J. Spero, Glacial–interglacial changes in Subantarctic sea surface temperature and δ^{18}O-water using foraminiferal Mg. *Earth Planet. Sci. Lett.* **170**, 417–432 (1999).

74. J. Groeneveld, C. M. Chiessi, Mg/Ca of *Globorotalia inflata* as a recorder of permanent thermocline temperatures in the South Atlantic. *Paleoceanography* **26**, PA2203 (2011).

75. R. A. Locarnini, A. V. Mishonov, O. K. Baranova, J. R. Reagan, T. P. Boyer, D. Seidov, Z. Wang, H. E. Garcia, C. Bouchard, S. L. Cross, C. R. Paver, D. Dukhovskoy, "World Ocean Atlas 2023, Volume 1: Temperature" (NOAA National Centers for Environmental Information, 2024).

76. M. Regenberg, A. Regenberg, D. Garbe-Schönberg, D. W. Lea, Global dissolution effects on planktonic foraminiferal Mg/Ca ratios controlled by the calcite-saturation state of bottom waters. *Paleoceanography* **29**, 127–142 (2014).

77. J. P. Marr, H. C. Bostock, L. Carter, A. Bolton, E. Smith, Differential effects of cleaning procedures on the trace element chemistry of planktonic foraminifera. *Chem. Geol.* **351**, 310–323 (2013).

78. M. Makarova, R. Tapia, M. Mohtadi, A. Hou, J. Groeneveld, A. N. Meckler, K. F. Huang, R. Y. Tung, Y. Iizuka, S. L. Ho, Contrasting scenarios for upper ocean temperature changes at the last glacial maximum inferred from inorganic and organic paleothermometers: A case study from the eastern tropical indian ocean. *Paleoceanogr. Paleoclimatol.* **40**, e2024PA004908 (2025).

79. H. Tandy, R. J. Flores, A. V. Subhas, D. N. Schmidt, K. T. Khan, S. Gwak, L. Savage, R. A. Eagle, A. Tripati, Dissolution effects on clumped isotope signatures in planktic foraminifera. *Paleoceanogr. Paleoclimatol.* **40**, e2025PA005113 (2025).

80. B. Hönisch, K. A. Allen, D. W. Lea, H. J. Spero, S. M. Eggins, J. Arbuszewski, P. deMenocal, Y. Rosenthal, A. D. Russell, H. Elderfield, The influence of salinity on Mg/Ca in planktic foraminifers – Evidence from cultures, core-top sediments and complementary $\delta^{18}O$. *Geochim. Cosmochim. Acta* **121**, 196–213 (2013).

81. A. N. Meckler, P. F. Sexton, A. M. Piasecki, T. J. Leutert, J. Marquardt, M. Ziegler, T. Agterhuis, L. J. Lourens, J. W. B. Rae, J. Barnet, A. Tripati, S. M. Bernasconi, Cenozoic evolution of deep ocean temperature from clumped isotope thermometry. *Science* **377**, 86–90 (2022).

82. N. Shackleton, "Attainment of isotopic equilibrium between ocean water and the benthonic foraminifera genus *Uvigerina*: Isotopic changes in the ocean during the last glacial," in *Colloques Internationaux du C.N.R.S.* (Centre National de la Recherche Scientifique, 1974), pp. 203–209.

83. H. Elderfield, P. Ferretti, M. Greaves, S. Crowhurst, I. N. McCave, D. Hodell, A. M. Piotrowski, Evolution of ocean temperature and ice volume through the mid-Pleistocene climate transition. *Science* **337**, 704–709 (2012).

84. D. Paillard, L. Labeyrie, P. Yiou, Macintosh Program performs time-series analysis. *Eos. Trans. AGU* **77**, 379–379 (1996).

85. L. E. Lisiecki, M. E. Raymo, Correction to "A Pliocene-Pleistocene stack of 57 globally distributed benthic $\delta^{18}O$ records". *Paleoceanography* **20**, PA2007 (2005).

86. J. Ullermann, F. Lamy, U. Ninnemann, L. Lembke-Jene, R. Gersonde, R. Tiedemann, Pacific-Atlantic Circumpolar Deep Water coupling during the last 500 ka. *Paleoceanography* **31**, 639–650 (2016).

87. L. Gregor, A. Fay, SeaFlux: Harmonised sea-air CO_2 fluxes from surface pCO_2 data products using a standardised approach, Zenodo (2021); https://doi.org/10.5281/zenodo.5482547.

88. Y. H. Park, T. Park, T. W. Kim, S. H. Lee, C. S. Hong, J. H. Lee, M. H. Rio, M. I. Pujol, M. Ballarotta, I. Durand, C. Provost, Observations of the antarctic circumpolar current over the udintsev fracture zone, the narrowest choke point in the southern ocean. *J. Geophys. Res. Oceans* **124**, 4511–4528 (2019).

89. J. Jouzel, V. Masson-Delmotte, O. Cattani, G. Dreyfus, S. Falourd, G. Hoffmann, B. Minster, J. Nouet, J. M. Barnola, J. Chappellaz, H. Fischer, J. C. Gallet, S. Johnsen, M. Leuenberger, L. Loulergue, D. Luethi, H. Oerter, F. Parrenin, G. Raisbeck, D. Raynaud, A. Schilt, J. Schwander, E. Selmo, R. Souchez, R. Spahni, B. Stauffer, J. P. Steffensen, B. Stenni, T. F. Stocker, J. L. Tison, M. Werner, E. W. Wolff, Orbital and millennial Antarctic climate variability over the past 800,000 years. *Science* **317**, 793–796 (2007).

90. F. J. Millero, A. Poisson, C. C. Tung, A. L. Bradshaw, K. Schleiker, “Background papers and supporting data on the International Equation of State of Seawater, 1980” (UNESCO, 1981).

91. J. R. Reagan, D. Seidov, Z. Wang, D. Dukhovskoy, T. P. Boyer, R. A. Locarnini, O. K. Baranova, A. V. Mishonov, H. E. Garcia, C. Bouchard, S. L. Cross, C. R. Paver, “World Ocean Atlas 2023, Volume 2: Salinity” (NOAA National Centers for Environmental Information, 2024).

92. B. Saulme, Sea Water - Equation of State Calculator - TEOS-10 & IES-80 (2013); https://monrecifamoi.saulme.fr/salinite/sea_water_calculator_teos10.php.

93. O. Orheim, B. Giles, G. Moholdt, J. Jacka, A. Bjørdal, The SCAR International Iceberg Database, Norwegian Polar Institute (2021); https://doi.org/10.21334/NPOLAR.2021.E4B9A604.

94. R. M. Key, A. Kozyr, C. L. Sabine, K. Lee, R. Wanninkhof, J. L. Bullister, R. A. Feely, F. J. Millero, C. Mordy, T. H. Peng, A global ocean carbon climatology: Results from Global Data Analysis Project (GLODAP). *Global Biogeochem. Cycles* **18**, GB4031 (2004).

95. A. Olsen, N. Lange, R. M. Key, T. Tanhua, M. Álvarez, S. Becker, H. C. Bittig, B. R. Carter, L. Cotrim da Cunha, R. A. Feely, S. van Heuven, M. Hoppema, M. Ishii, E. Jeansson, S. D. Jones, S. Jutterström, M. K. Karlsen, A. Kozyr, S. K. Lauvset, C. Lo Monaco, A. Murata, F. F. Pérez, B. Pfeil, C. Schirnick, R. Steinfeldt, T. Suzuki, M. Telszewski, B. Tilbrook, A. Velo, R. Wanninkhof, GLODAPv2.2019 – an update of GLODAPv2. *Earth Syst. Sci. Data* **11**, 1437–1461 (2019).

96. R. A. Locarnini, A. V. Mishonov, O. K. Baranova, T. P. Boyer, M. M. Zweng, H. E. Garcia, J. R. Reagan, D. Seidov, K. Weathers, C. R. Paver, I. Smolyar, "World Ocean Atlas 2018, Volume 1: Temperature" (NOAA Atlas NESDIS 81, 2018).

97. J. F. Adkins, K. McIntyre, D. P. Schrag, The salinity, temperature, and $\delta^{18}O$ of the glacial deep ocean. *Science* **298**, 1769–1773 (2002).

98. Jamovi Project, jamovi - open statistical software, Jamovi Project (2024); www.jamovi.org/.

99. D. W. Lea, T. A. Mashiotta, H. J. Spero, Controls on magnesium and strontium uptake in planktonic foraminifera determined by live culturing. *Geochim. Cosmochim. Acta* **63**, 2369–2379 (1999).

100. H. Elderfield, G. Ganssen, Past temperature and $\delta^{18}O$ of surface ocean waters inferred from foraminiferal Mg/Ca ratios. *Nature* **405**, 442–445 (2000).

101. C. Cléroux, E. Cortijo, P. Anand, L. Labeyrie, F. Bassinot, N. Caillon, J.-C. Duplessy, Mg/Ca and Sr/Ca ratios in planktonic foraminifera: Proxies for upper water column temperature reconstruction. *Paleoceanography* **23**, PA3214 (2008).

102. L. Jonkers, S. van Heuven, R. Zahn, F. J. C. Peeters, Seasonal patterns of shell flux, $\delta^{18}O$ and $\delta^{13}C$ of small and large *N. pachyderma* (s) and *G. bulloides* in the subpolar North Atlantic. *Paleoceanography* **28**, 164–174 (2013).

103. N. Vázquez Riveiros, A. Govin, C. Waelbroeck, A. Mackensen, E. Michel, S. Moreira, T. Bouinot, N. Caillon, A. Orgun, M. Brandon, Mg/Ca thermometry in planktic foraminifera:

Improving paleotemperature estimations for *G. bulloides* and *N. pachyderma* left. *Geochem. Geophys. Geosyst.* **17**, 1249–1264 (2016).

104. P. Anand, H. Elderfield, M. H. Conte, Calibration of Mg/Ca thermometry in planktonic foraminifera from a sediment trap time series. *Paleoceanography* **18**, 1050 (2003).

105. M. Regenberg, S. Steph, D. Nürnberg, R. Tiedemann, D. Garbe-Schönberg, Calibrating Mg/Ca ratios of multiple planktonic foraminiferal species with δ^{18}O-calcification temperatures: Paleothermometry for the upper water column. *Earth Planet. Sci. Lett.* **278**, 324–336 (2009).
